# DNA fingerprinting in botany: past, present, future

**DOI:** 10.1186/2041-2223-5-1

**Published:** 2014-01-03

**Authors:** Hilde Nybom, Kurt Weising, Björn Rotter

**Affiliations:** 1 Department of Plant Breeding–Balsgård, Swedish University for Agricultural Sciences, Fjälkestadsvägen 459, Kristianstad 29194, Sweden; 2 Plant Molecular Systematics, Institute of Biology, University of Kassel, Kassel 34109, Germany; 3 GenXPro GmbH, Altenhöferallee 3, Frankfurt 60438, Germany

**Keywords:** DNA fingerprinting, Genetic mapping, Genotyping-by-sequencing, Microsatellites, Plants, Population genetics, Single nucleotide polymorphisms, Systematics

## Abstract

Almost three decades ago Alec Jeffreys published his seminal *Nature* papers on the use of minisatellite probes for DNA fingerprinting of humans (Jeffreys and colleagues *Nature* 1985, 314:67–73 and *Nature* 1985, 316:76–79). The new technology was soon adopted for many other organisms including plants, and when Hilde Nybom, Kurt Weising and Alec Jeffreys first met at the very First International Conference on DNA Fingerprinting in Berne, Switzerland, in 1990, everybody was enthusiastic about the novel method that allowed us for the first time to discriminate between humans, animals, plants and fungi on the individual level using DNA markers. A newsletter coined “Fingerprint News” was launched, T-shirts were sold, and the proceedings of the Berne conference filled a first book on “DNA fingerprinting: approaches and applications”. Four more conferences were about to follow, one on each continent, and Alec Jeffreys of course was invited to all of them. Since these early days, methodologies have undergone a rapid evolution and diversification. A multitude of techniques have been developed, optimized, and eventually abandoned when novel and more efficient and/or more reliable methods appeared. Despite some overlap between the lifetimes of the different technologies, three phases can be defined that coincide with major technological advances. Whereas the first phase of DNA fingerprinting (“the past”) was dominated by restriction fragment analysis in conjunction with Southern blot hybridization, the advent of the PCR in the late 1980s gave way to the development of PCR-based single- or multi-locus profiling techniques in the second phase. Given that many routine applications of plant DNA fingerprinting still rely on PCR-based markers, we here refer to these methods as “DNA fingerprinting in the present”, and include numerous examples in the present review. The beginning of the third phase actually dates back to 2005, when several novel, highly parallel DNA sequencing strategies were developed that increased the throughput over current Sanger sequencing technology 1000-fold and more. High-speed DNA sequencing was soon also exploited for DNA fingerprinting in plants, either in terms of facilitated marker development, or directly in the sense of “genotyping-by-sequencing”. Whereas these novel approaches are applied at an ever increasing rate also in non-model species, they are still far from routine, and we therefore treat them here as “DNA fingerprinting in the future”.

## Fingerprinting plants in the past

### Telling plants apart in the olden days…

Many disciplines in botany are dependent on the ability to differentiate among plant genotypes, and/or to estimate the amount of diversity and relatedness in a set of genotypes. Traditionally, such tasks have been conducted mainly through data on morphological characteristics but these have certain limitations, including insufficient variation among the studied genotypes, subjectivity in the data collection and treatment, and plasticity due to environmentally induced variation. A more neutral and objective tool was offered by molecular markers based on isoenzymes; that is, enzymes that catalyze the same chemical reaction but differ in amino acid sequence and therefore also in the speed taken to travel through an electrophoretic gel. Isoenzymes were introduced into plant science in the early 1960s, and quickly increased in importance throughout the 1970s and 1980s. Co-dominant allozyme data (that is, allelic enzymes coded by genes at the same locus) soon became very popular for studies of, for example, population structure, gene flow, isolation-by-distance (IBD), mating systems and hybridization [1]. Protein extraction was, however, often a problem, especially for plants with high contents of polyphenols in their leaves. The analysis of plants growing in remote areas was also a problem, since proteins generally need to be isolated and purified within a short time from sampling. A third major problem was the often insufficient level of allozyme polymorphism among related genotypes.

Compared with proteins, the DNA molecule is very robust and easy to work with, and the potential for yielding polymorphic data is virtually inexhaustible. In the 1970s, the advent of the DNA-based restriction fragment length polymorphism (RFLP) technique enabled botanists to analyze samples collected from plants growing almost anywhere. Samples, usually leaves, were usually dried on silica gel before being transported to a laboratory, where they could be kept frozen until DNA isolation. The RFLP method was, however, rather time-consuming, with isolation of genomic DNA from the collected material, cutting the DNA samples with restriction enzymes, transferring the fragments with Southern blotting to a filter, hybridizing the filter-bound fragments with locus-specific probes, and finally utilizing for example autoradiography to detect the fragments. Still, the major constraint was the need for developing species-specific hybridization probes for these analyses. RFLP methodology was therefore applied mainly to economically important crop plants, with many active scientists and large grants. In these crops, RFLP markers constituted a highly appreciated tool for the development of genetic maps [2], and sometimes for cultivar identification and studies of genetic relatedness [3]. Nevertheless, a restricted availability of suitable loci often resulted in insufficient polymorphism also with the RFLP method.

In the 1980s, the RFLP methodology was also first applied to the chloroplast DNA (cpDNA) molecule. For this, DNA samples were digested with either single or combined restriction enzymes, and hybridized with radiolabelled cpDNA-specific probes from one of the universal libraries developed from, for example, *Petunia*. The obtained information was used to construct restriction site maps of the cpDNA molecule. Since the cpDNA molecule is highly conserved, there is very little intra-specific variation, and cpDNA-based RFLP studies have therefore mostly been conducted on an inter-specific level. By contrast, plant mitochondria have never been much used in molecular analyses. The major reason is that while the plant mitochondrial DNA (mtDNA) sequence is usually highly conserved, the size and structure of mtDNA molecules may vary widely even within individual plants [4]. Moreover, recent studies indicated that substitution rates of mtDNA genes can vary enormously even among closely related plant species [5].

### Minisatellite and oligonucleotide DNA probes enable true plant fingerprinting

When Jeffreys and colleagues [6,7] published their groundbreaking papers on RFLP analyses with probes developed from tandemly repeated DNA sequences in human DNA, nobody expected that this new, so-called DNA fingerprinting technique, would revolutionize also the botanical science. However, since these new minisatellite probes showed a high potential for revealing individual-specific DNA fingerprints also in other mammals [8] as well as in birds [9], botanists soon decided to investigate the possibilities of applying this tool also in plants. In a paper appearing in 1988, Dallas [10] was able to distinguish among different rice cultivars, *Oryza sativa*, by hybridizing restriction-digested rice DNA with the human 33.6 minisatellite probe. The studied offspring from an individual rice plant proved to have identical fingerprints, which is the expected result since rice is self-pollinating and thus highly homozygous. In addition, Dallas was able to ascertain the Mendelian inheritance of DNA fragments from grandparents to the second-generation offspring (F_2_).

In the same year, two more papers reported on fingerprinting plant material with another minisatellite probe, this time derived from the genome of the bacteriophage M13. Whereas Ryskov and colleagues [11] obtained different DNA fingerprint patterns of two barley varieties, *Hordeum vulgare*, after hybridization with the M13 probe, Rogstad and colleagues [12] generated identical M13 fingerprints from separate branches of a cottonwood tree, *Populus deltoides*, as well as from a mother tree and its sucker plant, demonstrating somatic stability. These authors also showed that fingerprints obtained from the offspring from an inbred tomato, *Solanum lycopersicon*, were identical, while a high level of variation was encountered among sexually derived cottonwood trees, indicating that the degree of variation depends on the mode of reproduction. In the following years, the ability of minisatellite probes to distinguish between specimens derived by sexual recombination and specimens derived by vegetative propagation or apomixis (that is, seed set without prior fertilization) was demonstrated in numerous plants, including North American quaking aspen, *Populus tremuloides*[13], and various raspberry and blackberry species, *Rubus* spp. [14,15].

In cultivated plants, propagation is undertaken either through seeds (especially in annual and biennial crops) or vegetatively (in fruit crops, as well as in many woody ornamentals). In the latter, each cultivar is expected to consist of a single monomorphic genotype. DNA fingerprinting thus became a very efficient means of investigating identity as demonstrated in some *Rubus* cultivars [16]. In such crops, new and unique characters sometimes appear in, for example, a single branch of a tree through the occurrence of minor somatic mutations. Propagation of material collected from these deviating plant parts gives rise to new cultivars (known as, for example, sports in apple). Analysis of sports marketed under different names but all derived from the well-known 'Red Delicious’ apple, however, produced completely identical DNA fingerprints [17]. Obviously none of the minor DNA differences between these sports had been targeted by the M13 probe.

**Figure 1:**
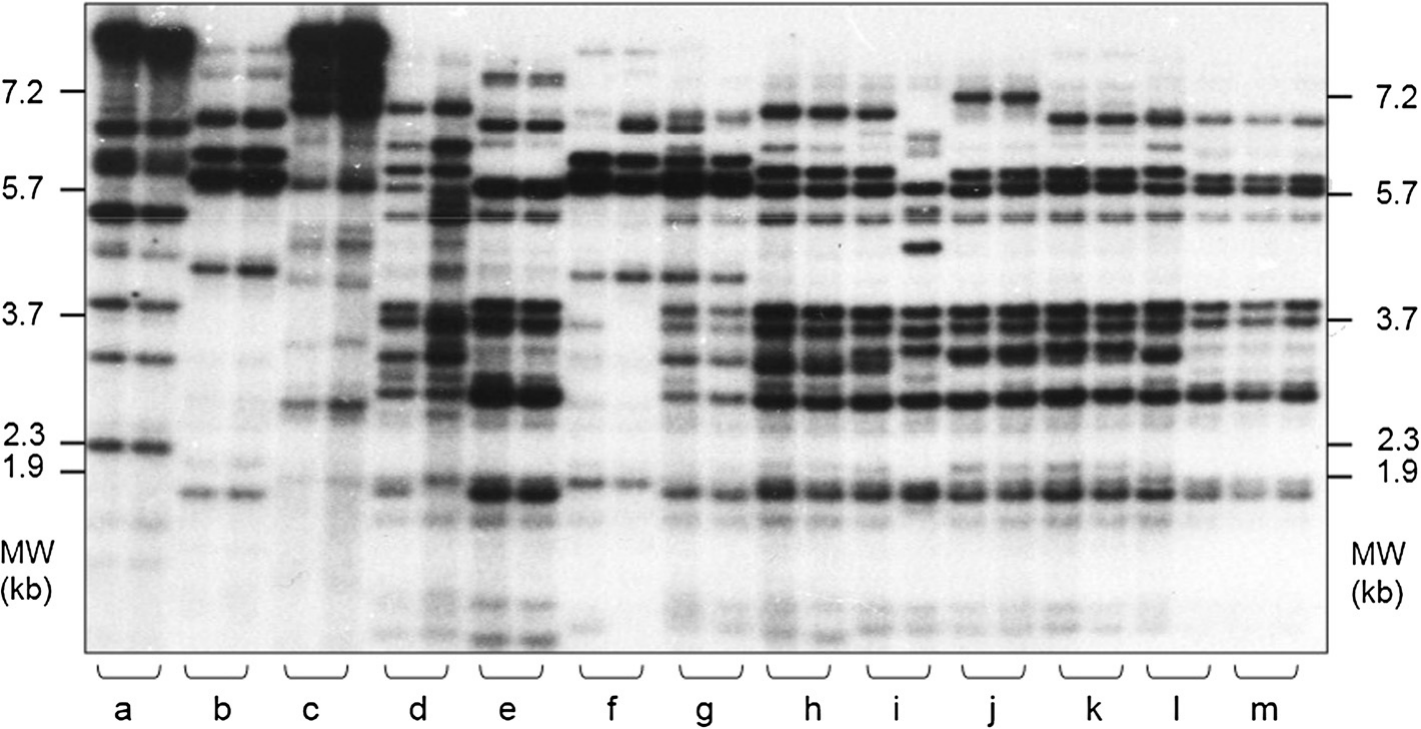
**Hybridization-based restriction fragment length polymorphism fingerprints of tomato plants (*Solanum lycopersicum*).** Five-microgram aliquots of genomic DNA from two individual plants each of three wild species (a to c) and 10 cultivars (d to m) were digested with the restriction enzyme *Hinf*I, separated on a 1.4% agarose gel, and in-gel hybridized with the radioabelled oligonucleotide probe (GGAT)_4_ (Kaemmer and colleagues [22]). Banding patterns were visualized by autoradiography. Positions of size markers are indicated (kb = kilobase pairs). MW, molecular weight.

Still another set of RFLP hybridization probes was introduced in the early days of DNA fingerprinting, namely synthetic oligonucleotides such as (GACA)_4_ and (GATA)_4_[18]. These probes hybridized to short, tandem-repeated sequences (microsatellites; simple sequence repeats (SSRs)) in the genome, and produced polymorphic fragment patterns in, for example, cultivars of chickpea, *Cicer arietinum*[19], banana [20], tomato [21,22] and rice [23], in double-haploid lines of sugar beet (*Beta vulgaris*) [24], and in wild plants of the Chilean annual *Microseris pygmaea*[25]. As an experimental bonus, the oligonucleotide probes allowed hybridization directly within the dried gels, thus circumventing Southern blotting altogether. A typical banding pattern resulting from this ancient, so-called “oligonucleotide fingerprinting” methodology is shown in Figure 1.

### Technical issues of hybridization-based plant DNA fingerprinting

The successful application of minisatellite and oligonucleotide probes for DNA fingerprinting by Southern blot hybridization is dependent on the availability of relatively large quantities of very clean DNA in order for the restriction enzymes to produce clear fragment profiles. DNA isolation thus became a crucial step, and many different protocols were developed [26,27]. Other methodological advances such as the use of non-radioactive fragment detection - for example, digoxigenin-based labelling - were also described [28,29]. Since oligonucleotide probes sometimes yield a high background and can be sensitive to minute changes in temperature during hybridization, a PCR-based method for producing longer and more robust probes (typically 300 to 600 bp) but still with short repeated motifs such as, for example, (GACA)_n_ was developed by Rogstad [30]. Hybridization of the same filter (stripped and re-hybridized consecutively) with nine of these so-called PCR-STR (synthetic tandem-repeat) probes produced polymorphic DNA fingerprints in turnip (*Brassica rapa*) plants, and allowed the verification of Mendelian fragment transmittal to the offspring [31].

Data evaluation remained relatively 'primitive’ in many of these early studies. Usually, the number of analyzed samples was well below 50, and numbers of polymorphic bands seldom reached more than 20 to 40 in each study. Moreover, experimentally induced differences in hybridization efficiency between the electrophoretic gels and filters often precluded the pooling of data from samples analyzed on different gels [32]. Therefore, manual comparisons of fragment profiles were usually performed for evaluating relationships among the studied samples. This information was then used to make deductions about, for example, the transfer of pollen among different cultivars in an apple orchard [33], the hybrid origin of a blackberry microspecies [34], and the mode of seed setting in experimentally produced blackberry hybrids [35]. For quantitative comparisons, the proportion of shared bands was usually calculated with Dice’s coefficient, also known as Nei and Li’s coefficient [36]. The results were compared with previous information on propagation and distribution of the investigated material. For example, Tzuri and colleagues [37] and Vainstein and colleagues [38] estimated variability among and within different groups of carnations, and obtained patterns that could be associated with mode of propagation (by seed or vegetatively) as well as the known origination (from *Dianthus caryophyllus* or from *Dianthus* hybrids). Analyses of genetic relatedness based on banding pattern similarity have been carried out also among, for example, *Rubus* cultivars [29,39] and among and within populations of wild plant species such as box elder, *Acer negundo*[40], and paw-paw, *Asimina triloba*[41].

Methods for assessing genetic diversity were soon improved, and reports on the use of DNA fingerprinting for estimating population genetics parameters, such as expected heterozygosity, Wright´s F-statistics and the number of migrants per population and generation, became increasingly common during the 1990s. Whenever tested, results obtained with these dominant multi-locus markers were usually consistent with those from previous studies using co-dominant allozyme markers. Using a resampling program, M13 fingerprinting-derived estimates for genetic identity within and between populations as well as population subdivision proved to be closely associated with the breeding system in three species of *Plantago*[42]. The selfing species *P. major* showed relatively little within-population variation compared to the mixed breeding *P. coronopus* and the outbreeding *P. lanceolata*. Interpopulation differentiation was, by contrast, more pronounced in the selfing species compared with the other two. Population genetics parameters from RFLP-based fingerprinting data were reported also in, for example, Gambel oak, *Querus gambelii*[32], common cattail, *Typha latifolia*[43], and two species of buckeye, *Aesculus*[44].

For more information on the methodology, applications and results obtained by hybridization-based fingerprinting with mini- and microsatellite complementary probes, see the reviews by Nybom [45,46], Weising and colleagues [26], Rogstad [47] and Weising and Kahl [48].

## Present-day fingerprinting of plants

### Method development and choice of markers

#### PCR-based multi-locus methods

**Figure 2:**
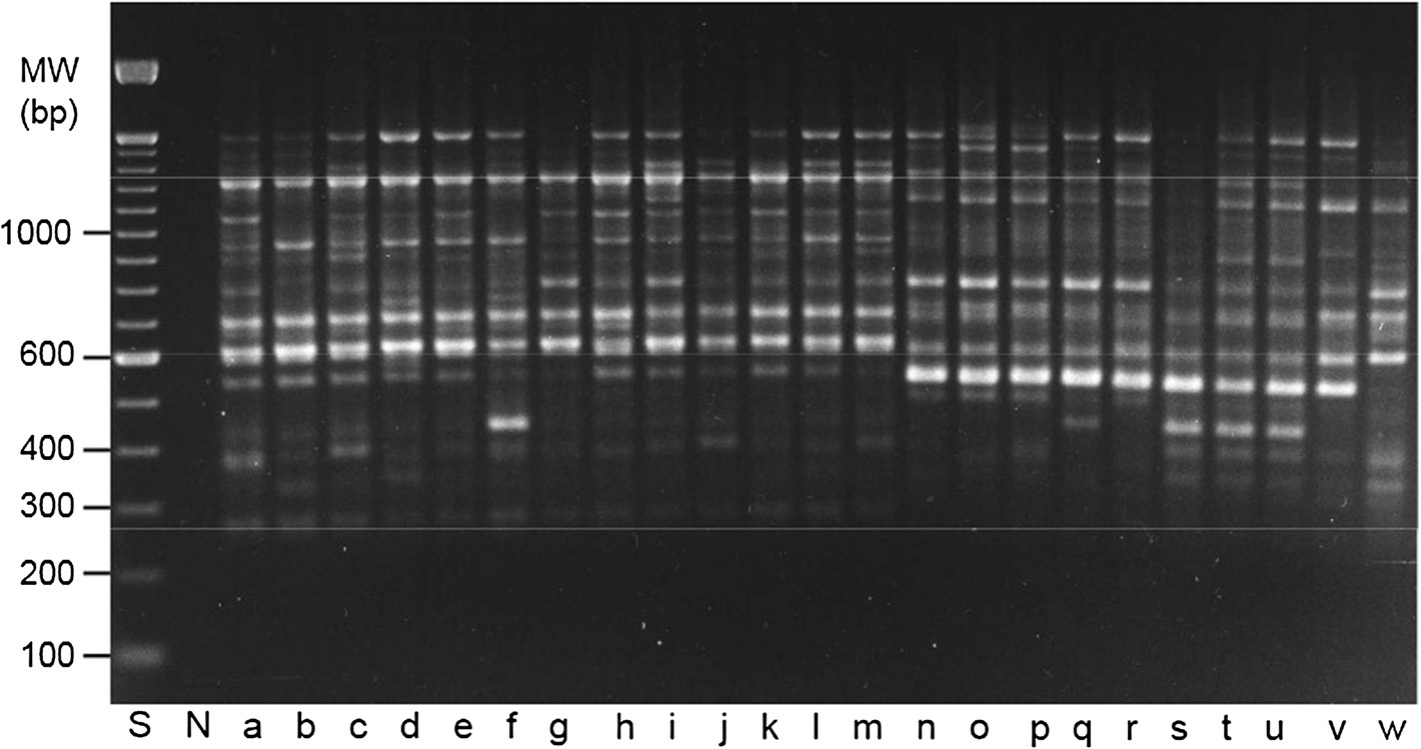
**Random amplified polymorphic DNA (RAPD) fingerprints of *Pelargonium*.** DNA aliquots from 13 cultivars of *Pelargonium hortorum* (lanes a to m), nine cultivars of *P. peltatum* (lanes n to v), and one individual of the wild subspecies *P. peltatum* ssp. *dibrachya* (lane w) were amplified with the arbitrary 10-mer primer OPG-5. RAPD products were separated on a 1.5% agarose gel and stained with ethidium bromide. Positions of size markers (lane S) are indicated in base bairs (bp). N, negative control (no template DNA in the PCR assay). MW, molecular weight.

Shortly after the invention of the ingenious PCR procedure by Saiki and colleagues [49], three PCR-based approaches to generate DNA fingerprints were published more or less at the same time. All of these methods used single oligonucleotide primers with arbitrary sequences to produce PCR fragments from genomic DNA, resulting in multi-locus banding patterns after electrophoretic separation and visualization by staining or radiography [50-52]. The so-called random amplified polymorphic DNA (RAPD) approach developed by Williams and colleagues [51] soon became the most popular variant of these methods. Major explanations for this immediate success include the small quantities needed of sample DNA, and the simple and fast procedures compared to the hybridization-based methods. Results from a typical RAPD experiment are illustrated in Figure 2.

A few years later, Zabeau and Vos [53] and Vos and colleagues [54] presented the amplified fragment length polymorphism (AFLP) technique, which represented an ingenious combination of RFLP and PCR methodology. AFLP analyses became soon very popular, mainly because of the large numbers of polymorphic bands obtained in a single experiment. The inter-simple sequence repeat (ISSR) method developed by Gupta and colleagues [55] and Zietkiewicz and colleagues [56] relied on microsatellite-complementary PCR primers that could be used in an anchored or unanchored version. RAPD, AFLP and ISSR are still much used nowadays, although RAPD especially has often been criticized for problems with reproducibility and competitive priming, as reviewed in Weising and colleagues [26]. These problems are less pronounced for AFLP and ISSR where more stringent PCR conditions can be applied. Nevertheless, all three methods usually arrived at quite similar estimates of genetic diversity and genetic distances when applied to the same plant material, as reviewed in Weising and colleagues [27].

Other less frequently used methods to generate multi-locus PCR fingerprints include the sequence-related amplified polymorphism (SRAP) technique that specifically amplifies polymorphic junction fragments between exons and the flanking intronic DNA [57,58], and the target region amplification polymorphism (TRAP) method [59]. Common features of SRAP and TRAP include the use of two primers of about 18 nucleotides length (one of which targets a protein-coding region), and non-stringent PCR conditions during the first five cycles. The so-called selective amplification of polymorphic microsatellite loci (SAMPL) is a variant of the AFLP technology that combines AFLP- and microsatellite-specific primers [60], whereas the direct amplification of minisatellite DNA (DAMD) utilizes primers that are specific for minisatellites rather than microsatellites [61]. Yet another approach, resistance gene-analog polymorphism (RGAP), makes use of PCR primers that bind to the conserved domains of plant resistance genes [62].

The Diversity Arrays Technology (DArT) is a high-throughput method based on the hybridization of fluorescent DNA probes to a set of target DNAs spotted onto a microarray [63,64]. The DNA is first digested with one or two restriction enzymes, followed by the ligation of specific adapters as in AFLP. Individual PCR products are spotted onto a grid to form an ordered microarray that represents hundreds of arbitrarily selected restriction fragments from all cultivars/species and various genomic regions of the gene pool of interest. Individual genomic DNA samples are pretreated in the same way as the pooled representatives (that is, restriction, ligation of adapter, and PCR with adapter-specific primers). Before being individually hybridized to the chip, each probe DNA is labelled with a fluorochrome to enable detection. Like AFLP and RAPD, DArT does not require previous sequence information. It allows simultaneous analysis of hundreds or even thousands of polymorphic loci, but the need to generate a microarray restricts the general use of the technique. By 2012, DArT technology has been developed for about 60 organisms, mostly crop and model plants [65], but also some wild plants such as the fern *Asplenium viride*[66].

Transposable elements and especially the retrotransposons bounded by long terminal repeats (LTRs) have proved to be useful for developing particularly sensitive multi-locus profiling techniques, either alone or in combination with other types of primers [67]. In the inter-retrotransposon amplified polymorphism (IRAP) approach developed by Kalendar and colleagues [68], primers are directed towards the LTRs of *BARE*-1, a retrotransposon of barley. The same authors also introduced retrotransposon-microsatellite amplified polymorphism (REMAP) which combines outward-facing LTR-specific primers with anchored microsatellite primers. Basically the same strategy, known as *copia*-SSR, was simultaneously developed by Provan and colleagues [69]. In the so-called sequence-specific amplification polymorphism (S-SAP) analysis, retrotransponson-specific primers are combined with AFLP primers [70]. S-SAP often produces highly variable fingerprints that are frequently more informative than AFLP. Related approaches have been developed for other plant transposons [71,72].

#### PCR-based single-locus methods

**Figure 3:**
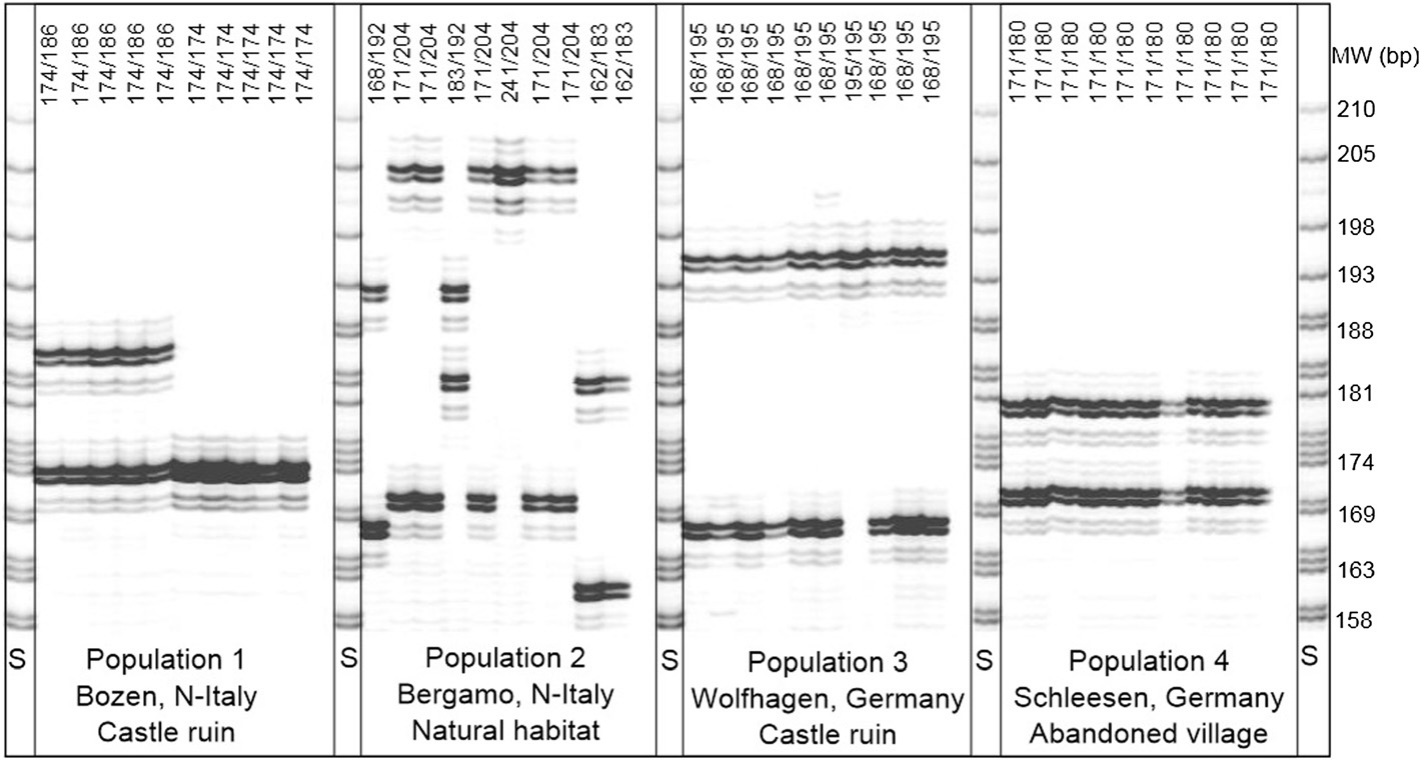
**Locus-specific microsatellite analysis of four populations of the Lesser Periwinkle (*Vinca minor*) using a primer pair specific for locus Vimi43 (Möller, personal communication).** For each population, ten samples were genotyped. Populations 1 and 2 were collected in northern Italy, whereas populations 3 and 4 were sampled in central Germany. Strong indications for clonality can be found in populations 1, 3 and 4. S, size standard: T-ladder derived from a chloroplast DNA fragment of *Macaranga indistincta*. Molecular weights (MW) of size markers are indicated in base pairs (bp).

Because of their abundance, high polymorphism in the number of tandem repeats, co-dominant inheritance, excellent reproducibility and ease of use, PCR-amplified single-locus microsatellite markers have become the marker of choice for many applications, and presently remain more important than any of the other traditional DNA fingerprinting methods [73,74]. Typically, a pair of microsatellite-flanking primers is used to amplify the targeted locus by PCR, amplification products are separated by polyacrylamide or capillary electrophoresis, and banding patterns are monitored by radiography or fluorography. When locus-specific microsatellite analysis was first used in plants in 1992 [75], the need for developing species-specific microsatellite-flanking primers was still a serious drawback, requiring tedious cloning and enrichment strategies (see the reviews by Squirrell and colleagues [76] and Weising and colleagues [27]). Nowadays, this task has become relatively simple for (1) the increasing number of plant species with DNA sequence data in public databases and (2) the development of ultrafast “next generation sequencing” technologies that enable the identification of microsatellite loci and design of primers by random genomic sequencing (see "The future of DNA fingerprinting" below). A typical result from a microsatellite genotyping experiment is shown in Figure 3.

Lately, expressed sequence tags (ESTs) have become a viable alternative to genomic DNA as a source for SSR loci, resulting in so-called EST-SSR markers that are either generated by cDNA cloning and sequencing [77] or, more commonly, by exploiting existing EST databases [78-80]. Database mining is often quite efficient, since EST-SSRs are surprisingly common and may be expected every 2 to 10 kb of EST sequence; for example, one per 6.3 kb in *Hordeum vulgare*[78]. These estimates of course depend on the search criteria and the search script used, most importantly on the minimum number of repeats used to define a microsatellite. Trinucleotide repeats commonly prevail in protein-coding regions of ESTs, whereas dinucleotide repeats are more frequent in 5′ and 3′ untranslated regions (UTRs). Expansions and deletions in coding regions can be tolerated for tri- and hexanucleotide repeats, because they do not perturb reading frames.

EST- and cDNA-derived SSRs have several important advantages over anonymous markers (see the review by Varshney and colleagues [81]). First, developing markers from already existing sequences is easy, fast and economical. Second, any type of microsatellite will be detected, whereas only SSRs with predefined motifs are captured by enrichment strategies. Third, EST-SSRs are physically linked to an expressed gene, which may encode a trait of interest. Finally, primer target sequences that reside in transcribed DNA regions are expected to be relatively conserved thus enhancing the chance of marker transferability across taxa. On the negative side, the association with coding regions sometimes limits the polymorphism of EST-derived SSR markers, resulting in fewer alleles and/or lower observed heterozygosity [78], but this is not necessarily the case. For example, Pashley and colleagues [79] compared the performance of 48 anonymous versus 48 EST-derived SSR markers from common sunflower, *Helianthus annuus*, and their transferability to two other *Helianthus* species. Their study showed that: (1) 73% of the EST-derived SSR markers were transferable among all species, compared with only 21% of the anonymous SSR markers; (2) EST-SSRs were on average only slightly less polymorphic that anonymous SSRs, both in the focal and the non-focal species; and (3) EST-SSRs located in coding regions were more readily transferable than those in untranslated regions - without differing significantly from the latter in terms of variability.

Locus-specific markers can also be developed from individual bands within multi-locus profiles, as exemplified by the sequence characterized amplified regions (SCARs). In the original description of the approach, specific primer pairs were designed for amplifying single bands of a RAPD profile [82]. SCARs have been used for cultivar identification in, for example, olive, *Olea europaea*[83], and sweet cherry, *Prunus avium*[84]. In the cleaved amplified polymorphic sequences (CAPS) approach [85], the resulting PCR product is treated with a restriction enzyme before scoring of fragments.

#### Single nucleotide polymorphisms

In the last decade, DNA fingerprinting methods based on single nucleotide polymorphisms (SNPs) have become increasingly important, especially in conjunction with microarray analyses that allow the simultaneous screening of very large numbers of SNP sites (see the review by Appleby and colleagues [86]). Among the many types of mutations occurring in genomes, single nucleotide exchanges stand out by their high absolute numbers as well as their biallelic nature, relatively low mutation rates, even distribution across the genome and relative ease of detection. In plants, one SNP is typically found per approximately 100 to 500 bp of DNA, but the average density depends on the studied species and the genomic region investigated. Numerous technologies have been developed for SNP discovery as well as for SNP genotyping [86]. Direct sequencing of multiple copies of the same genomic region is the most obvious method for SNP discovery, and has become very efficient after the development of high-throughput sequencing systems [87,88] (see "The future of DNA fingerprinting" below). Like SSRs, SNPs can also be mined from existing databases [89]. Practically all SNP genotyping assays are amenable to automation and therefore allow routine high-throughput analyses of large numbers of samples.

SNP markers are already well established in all major crop species [90,91], especially in those for which fully sequenced genomes are available. Recently, microarrays with typically 10,000 to 40,000 SNP markers (SNP-Chips) have been developed for many crops, and large-scale screenings of germplasm collections can now be undertaken at comparatively low costs [92]. Given that thousands of SNPs can be detected by novel sequencing approaches, SNP genotyping will receive increased attention, at least in economically important crop plants. However, poor transferability to related species may hamper their successful implementation for large-scale genotyping projects across genera.

#### Organellar DNA-based methods

The most commonly used organelle for genetic studies in plants is the chloroplast. Since recombination is rare or absent in plastid genomes, all DNA polymorphisms for a certain individual can be combined to form a “haplotype”. Plastid DNA polymorphisms at the intra-specific level are relatively rare, and the numbers of detected band profiles (haplotypes) are therefore often considerably lower than those detected by nuclear markers. On the positive side, the high conservation of organellar DNA sequences has enabled the development of non-specific, so called universal, PCR primers that amplify cpDNA introns and intergenic spacers in a wide array of plant species [93,94]. Universal primers are also available for the amplification of SSR loci in the chloroplast genome [95]. Polymorphisms within the amplified fragments can be monitored by various approaches, including the detection of length variants by high-resolution electrophoresis, and the detection of sequence variants by sequencing, or by digesting the PCR products with restriction enzymes in an approach called PCR-RFLP.

Plastid DNA is especially useful in studies where a low mutation rate is desirable, such as in the analyses of phylogenetic and phylogeographic patterns. Often both plastid and nuclear markers are combined in the same study for complementary information. Since the mode of plastid inheritance is usually maternal in angiosperms and paternal in gymnosperms, these markers also have the potential for tracing uni-parental lineages over large distances in time and space.

#### Choice of method

The pros and cons of different molecular marker methods have been discussed in a number of comparative investigations (see [27,96]). The actual choice of method must of course take marker availability, costs, expertise, equipment and many other factors into consideration. Based on 292 papers published between mid-2006 and mid-2009 on discrimination among plant cultivars, locus-specific microsatellite analysis (SSR) was the most popular method (36%), followed by RAPD (27%), ISSR (13%), AFLP (11%), other nuclear DNA-based methods (10%, including for example CAPS, DAMD, IRAP, REMAP, SNPs, SCAR and SRAP) and organellar DNA-based methods (3%, mostly cpDNA) [97]. If the purpose of a study is to simultaneously discriminate both dissimilar and very similar entities, applying a whole battery of marker types may be the best solution.

While insufficient repeatability of DNA marker profiles can be regarded as a methodological artefact, insufficient germline stability of sequences corresponding to DNA markers can cause “biological artefacts” due to excessively high mutation rates. This problem is most likely to arise with the most sensitive types of markers, such as SSRs. The ability to merge data from different studies, even when developed in different laboratories, is a major asset of this method. The same is true for the other single-locus DNA markers, such as SNPs, SCARs and CAPS, but these are usually only biallelic. Nevertheless, the potential number of SNPs is virtually unlimited, and various SNP-based assay methods have already been developed (see above). In a comparative study on 58 maize inbred lines, SNPs outperformed SSRs both in terms of quality and quantity [98].

Exceptionally high mutation rates and reduced germline stability are often encountered when using retrotransposon-based markers [67]. Thus, several reports have indicated that S-SAP markers are especially useful for discriminating among clones derived by somatic mutations [99] or among genotypes derived by recombination among highly similar entities [100]. The S-SAP primers are usually designed according to species-specific sequence information but positive results have also been obtained by using universal retrotransposon-based sequences [101].

Besides their application for the identification of plant material and for the estimation of similarity and relatedness, DNA markers have been extensively used for the construction of genetic linkage-based genomic maps, with a major aim of identifying markers that are closely linked and therefore co-inherited with genes for specific traits (see "Genetic mapping" below). Dense linkage maps have been constructed for numerous plant species including all major crops using all kinds of markers. For ease of scoring when screening large numbers of progeny, singe-locus biallelic markers such as SNPs are usually preferred for this purpose.

### Applications of present-day DNA fingerprinting in plants

#### Genotype identification

Since the humble beginnings in 1988, DNA fingerprinting has become an immensely important instrument for genotype identification in both wild plant species and their cultivated relatives. Plants differ widely in life history traits including reproductive parameters such as propagation method and, for those that propagate by seed, also in breeding system (selfing or cross-pollinated) and in the mode of pollen and seed dispersal. All these factors have profound influence on the amount and partitioning of genetic variability between and within various entities such as cultivars and populations. These differences affect the utilization of DNA markers for fingerprinting individual plants or genotypes.

In some cases, DNA-based estimates of similarity among a set of genotypes show a relatively close association with previous morphology-based estimates, but there are also considerable discrepancies in other cases. If the morphological characters are mostly quantitative in nature, correspondence with DNA marker estimates is generally quite high as compared to qualitative characters, which are more likely to reflect only a small number of mutation events. It has also been suggested that molecular data are better at differentiating cultivated genotypes as well as their wild relatives according to origin and pedigree, whereas conventional pomological characterization data are more closely associated with physiological properties [102].

##### Genotype identification in wild plants: the influence of life history traits

Proper identification of individual genotypes is an important basis for many wild-plant-based studies. As mentioned above, various life history traits affect the amount and partitioning of genetic variation. Inbreeding species are, for example, most useful for forensic applications, since they typically produce suitable-sized patches of genetically identical or almost identical plants [103]. By contrast, outcrossing species are characterized by a situation where every plant has a different genotype. While potentially very informative, it is usually extremely difficult to secure forensic evidence involving a particular, unique plant specimen. Clonal plants, whether due to extensive vegetative propagation or apomixis frequently produce large numbers of progeny with the same genotype (see also Figure 3). Such genotypes can cover large geographical areas and are thus not sufficiently accurate for tying botanical evidence to a certain location.

In other research areas, the variation in plant life history traits can, however, be regarded as a positive factor; a wide range of biological questions can be answered by choosing suitable material and methods. DNA marker analyses have thus been able to estimate genotype age in plant clones, which have often proved to be considerably larger - and therefore often also older - than expected from previous data. For example, Steinger and colleagues [104] studied *Carex curvula*, a sedge species found in the European Alps. RAPD analysis of 116 tillers from a small patch (2.0 × 0.4 m) identified a total of 15 multi-locus genotypes. More than half of the sampled tillers proved to belong to a single, large clone estimated to be around 2,000 years old. Invasive species sometimes produce particularly large clones, such as the Japanese knotweed, *Fallopia japonica*, and the alligator weed, *Alternanthera philoxeroides*, both of which displayed a single RAPD phenotype in spite of being sampled over very large areas [105,106]. In other cases, DNA marker analyses have revealed more heterogeneity than expected. Each of five investigated Chinese populations of the invasive water hyacinth *Eichhornia crassipes* were thus shown to consist of at least three different clones according to their RAPD profiles [107].

Information about clonal growth can be very helpful for determination of factors involved in shaping population structure. When a microsatellite-based study was carried out in the marine eelgrass *Zostera marina*, clonal size proved to be positively correlated with heterozygosity [108]. Outbreeding clones were larger and contained more flowering shoots, indicating that inbreeding depression had decreased vigor and fertility. An unexpectedly high degree of genetic homogeneity was recently described in the geophyte *Gagea spathacea*[109]. All but two of 138 examined specimens, representing 52 populations throughout the entire distributional area in northern, central and eastern Europe, had identical AFLP profiles. Probably this highly polyploid taxon has derived from a hybridogenic event, and has managed to attain its large area almost exclusively by bulbil production and spreading rather than by seed set and seedling establishment.

DNA fingerprinting has also helped to clarify the reproductive system in species that can produce seeds both sexually and asexually (that is, by apomixis). Many *Taraxacum* populations consist of triploid individuals that apparently reproduce through apomixis and therefore are clonal. Such clones can sometimes cover large areas as was demonstrated in an AFLP study [110]. A comparison of SSR and AFLP data showed that both marker types were able to discriminate among nine apomictic microspecies (defined on morphological characteristics) of *Taraxacum*, but that AFLP was more sensitive in detecting also small, mutation-derived differences within each microspecies [111]. By contrast, two dinucleotide repeat SSR loci detected considerably more variation than AFLP in apomictic lineages of *Ranunculus carpaticola*[112,113]. Evidence for an origin by mutation instead of by recombination was provided by the lack of allele segregation in the investigated SSR loci. Thus, in each lineage, the same number of alleles was always found within a locus, and these alleles also formed classes of related allele sizes within each lineage.

The availability of adequate tools to identify individual genotypes can be immensely useful in plant ecology. Thus, SSR-analysed *Taraxacum* clones were recently employed to investigate biodiversity and ecosystem functioning. In one study, five identified *Taraxacum* clones were used for setting up experimental plots where effects of levels of diversity in both favorable (fallow field) and unfavorable (mowed lawn) conditions could be quantified [114]. The genotypic diversity effects appear to be stronger in environments where intra-specific competition is more intense. In a parallel study, genotype × environment associations were studied in natural populations with the same set of *Taraxacum* clones [115]. Genotypes that produced poorly under favorable conditions instead showed the highest performance under stressful conditions.

##### Genotype identification in vegetatively propagated cultivars

All plants belonging to a particular cultivar of an asexually propagated crop are expected to share identical DNA fingerprints, except for rare mutations. By contrast, sexually derived cultivars are expected to exhibit non-uniform fingerprint patterns. However, there are crops for which the major breeding method involves selection amongst rather similar seedlings that originate from a very small number of widespread cultivars. This situation can be exemplified by peach, *Prunus persica*, which is self-fertile and self-pollinating to a large extent. In such crops, new cultivars sometimes have DNA fingerprints that are almost identical or at least very similar to those of the seed parent in spite of being derived through sexual recombination. By contrast, variation is sometimes encountered where one expected uniformity. Vegetatively propagated crops are usually still capable of producing sexually derived seeds, and these may germinate and develop into fertile but unnoticed plants in less well-tended fields and orchards. There is therefore an increased risk, especially for older cultivars, that a certain name is being used on several different entities, some of which have originated from seed setting.

Irrespective of propagation and breeding method, the value of accessions in plant genetic-material collections benefits tremendously from DNA marker-aided identification. This is, however, especially important in vegetatively propagated crops that must be grown in the field or maintained in greenhouses at high costs. Previous reviews [97,116] show that a higher number of mislabelled plant accessions are revealed using DNA markers (typically 25 to 30% mislabellings) as compared to traditional (pomological or ampelographic) characters (typically 5 to 10% mislabellings). Different categories of problems with synonyms and homonyms have been defined [116], and appear to be especially prevalent in locally grown and often older germplasm whereas well-known modern-day cultivars are correctly identified to a much higher extent.

For large-scale profiling of, for example, accessions in a genetic resources collection, SSR markers are usually preferred [117]. Although generally regarded as highly reproducible, problems are sometimes encountered with incorrect allele sizing, the occurrence of null alleles, allele drop-out (only one of two alleles is amplified at a heterozygous locus), false alleles (artefactual amplification products) and occasional amplification of isoloci (an isolocus is a similar but non-identical locus in the genome, common in allopolyploid species). While dinculeotide repeat SSRs are the most common type of microsatellite, less stuttering and improved allele sizing can be achieved with markers based on tri- or tetranucleotide repeats [117,118], although these are sometimes also less informative.

Zhang and colleagues [119] checked the accuracy and reliability of 15 SSR loci for clone identification in cacao, *Theobroma cacao*, and reported an average error rate of only 0.014 for allele drop out and 0.019 for false alleles. Some loci were more error-prone than others, suggesting that putative loci should be evaluated not only for their polymorphism but also for reliability prior to large-scale analyses. Vélez and Ibánez [120] checked 19 SSR loci in a study of more than 4,000 plants representing 19 grapevine cultivars. After removal of some minor technical artefacts, 99.8% of the samples matched to the expected genotype. Some loci, however, proved to be rather sensitive to the occurrence of chimeric mutations whereas others were not. Artefactual variation of SSR markers was also indicated in a study of olive [121]. Interestingly, SSR alleles that differed among olive samples from the same cultivar were only 2 bp (one repeat unit) apart, whereas samples from different cultivars usually exhibited larger size dissimilarity in the polymorphic alleles. If available, accurate pedigree information is very valuable for checking the reliability of marker profiles.

The possibility to merge SSR-derived data from different investigations is often reported as a major asset of this method. This option is, however, dependent on the use of identical SSR loci and suitable standardization procedures. Since absolute allele sizes of the SSR markers often differ when results from different laboratories are compared, a representative reference material with many different alleles should be used at all laboratories involved in the genotyping program, and the material for these standards should be harvested from predetermined plants in one collection only. By comparison with suitable standard alleles, sample alleles can then be defined according to relative number of core repeat units instead of relying only on the absolute fragment length in base pairs.

Increased attention has recently been paid to the use of SNP markers for genotype identification in vegetatively propagated cultivars. Advantages of SNPs are their potential abundance and the fact that they do not rely on fragment length variation like SSR, and therefore are easier to standardize across different laboratories and equipment. Numerous high- and low-density SNP arrays have recently been developed for different crops. For example, a set of 48 SNPs was developed in grapevine, *Vitis vinifera*, through resequencing of 11 genotypes [122]. High-throughput SNP genotyping can be conducted using bead arrays or microarrays (SNP chips) such as in, for example, *Citrus*[123]. Since the number of polymorphisms covered in these assays is usually several hundred to many thousand, the obtained data can also be used for detecting quantitative trait loci (QTL).

##### Genotype identification in seed-propagated cultivars

In seed-propagated crops, at least some genetic variation usually persists also within cultivars. This is especially pronounced in highly outcrossing species, thus making DNA-marker-aided cultivar identification considerably more difficult. The situation is further complicated by the fact that each seed production cycle can lead to the introduction of new genetic variation - for example, due to foreign pollen. A considerable influx of new alleles was thus demonstrated after 7 to 13 subsequent regenerations of open-pollinating rye, *Secale cereale*[124].

Even with all the precautions taken in connection with modern gene bank regenerations, changes in allele frequencies can result from just recombination and selection. This was clearly demonstrated in an AFLP analysis of 50 white cabbage, *Brassica oleracea*, accessions together with first-generation regeneration products from six of these accessions [125]. The genetic changes between original accessions and their respective regenerants were of the same magnitude as the differences among some of the more similar accessions. Moreover, while most alleles remained stable between generations, frequencies of some alleles instead changed considerably, suggesting that unintentional selection had taken place.

Obviously, a large number of markers are required for proper quantification of genetic changes between generations, and for efficient discrimination among outcrossing, seed-propagated cultivars. Such large numbers are, for example, provided by the DArT technology that proved very useful for distinguishing *Festulolium* cultivars (*Festuca* × *Lolium* experimentally produced hybrids) with 7,680 probes on a microarray [126]. In this study, each cultivar was represented by 20 individual plants. These plants were analyzed both as individual and bulked samples. In order to minimize the loss of low-frequency bands, bulks with only five plants in each were recommended.

Inbreeding crops are usually considered less problematic than outcrossers, since the cultivars are more homogeneous. However, some inbreeding crops still contain intra-varietal variability, especially in the case of primitive cultivars or landraces. Propagation cycles performed in a genebank with such material can cause prominent gene frequency changes due to gene flow and inadvertent selection. In these cases, pure-lining of the accessions may be necessary to avoid loss of diversity, as exemplified by the USDA Soybean Germplasm Collection [127]. In addition, selfing crops often contain a multitude of genetically very similar cultivars, thus necessitating the use of highly polymorphic markers for discrimination. While the commonly applied AFLP and SSR markers have produced sufficient results in many studies, the retrotransposon-based S-SAP method has been shown to resolve even very closely related plant accessions in, for example, wheat, *Triticum aestivum*. Nowadays, SNP markers receive increasing attention also in sexually propagated crops, mainly for the almost inexhaustible number of potential polymorphisms. Genome-specific SNPs have thus been developed from wheat gene intronic regions, and have proven highly useful for cultivar discrimination as well as enabling a quantification of genetic diversity at each of the genomes in this hexaploid crop [128].

##### Genotyping somatic mutations

Spontaneously occurring somatic mutations can give rise to so-called 'sports’. These deviate from the original cultivars in minor but economically important traits such as fruit color in fruit and berry crops, and flower or leaf color in ornamentals. Sports are difficult to distinguish with DNA fingerprinting since the markers usually cover only a minute part of the genome. In addition, chimeras are quite common - that is, mutations that occur in only one of the three meristematic cell layers in the apical meristem that differentiate into the various plant tissues. The existence of chimerism was very elegantly demonstrated in grapevine, *Vitis vinifera*, by Franks and colleagues [129]. Although grapevine is a diploid species, some SSR loci occasionally showed three alleles when different sports were analyzed. It turned out that plants regenerated from cell layers L1 and L2, respectively, had different SSR alleles as well as different phenotypic characteristics. SSR analysis was used to identify chimeric clones also in 'Cabernet Sauvignon’ [130], 'Grüner Veltliner’ [131] and 'Moscatel Galego Branco’ [132] while clones of 'Pinot’ were successfully distinguished with the S-SAP method [99]. In this study, three different retrotransposon-based primer pairs produced a total of 1,274 bands, one third of which were polymorphic and able to discriminate among all the 19 investigated clones.

S-SAP analysis has been successful for the genotyping of sports also in other crops, such as apple. Using 15 S-SAP primer combinations, five sports of 'Gala’ and one of 'Braeburn’ could be discriminated, both from each other and from the two original genotypes [133], whereas 24 SSR primer pairs generating a total of 64 alleles, and 35 AFLP primer combinations generating more than 1,000 bands, failed to do so. Based on two *Ty1*-*copia* LTR retrotransposons, a set of 19 bud sports of the apple cultivar 'Fuji’ were investigated with S-SAP [134]. All sports obtained unique DNA profiles. Other retrotransposon-based methods can also be quite useful. In 24 sports of clementine, *Citrus reticulata*, application of eight IRAP primers produced a total of five polymorphic bands whereas RAPD (26 primers), ISSR (16 primers), AFLP (8 primer combinations), S-SAP (9 primer combinations) and SSR (9 primer pairs) revealed, at the most, one (S-SAP) or two (RAPD) polymorphisms [135,136].

Some studies found surprisingly high levels of marker polymorphism within cultivars, such as in olive, where clones have been selected and subsequently multiplied by vegetative propagation for centuries. In one study, 27 putative clones of 'Verdeal-Transmontana’ could be differentiated with ISSR [137] while even higher levels of polymorphism were encountered with RAPD (50% polymorphic bands) and ISSR (54%) in the screening of 120 putative clones of 'Cobrançosa’ [138]. Possible explanations for these observations include a polyclonal origin, accumulation of somatic mutations over the long life-span of this woody species, and unnoticed establishment of sexual progeny in the orchards.

##### Genotyping in vitro-propagated material

Heritable somaclonal variation - that is, variation among regenerants due to somatic mutations - can be significantly enhanced by some micropropagation techniques. Although often regarded as an undesirable side-effect, these mutations can be valuable in crops that lack sexual reproduction (such as, for example, banana) or have very long generation cycles (such as, for example, palm trees). In general, axillary branches yield the most stable regenerants, followed by somatic embryogenesis and finally organogenesis. It is, however, impossible to predict whether markers will be able to find any variation in regenerated material, or what methods will prove to be most efficient.

Very few polymorphisms have generally been found in tissue culture regenerants. The extent of DNA marker polymorphism can, however, vary considerably between plant materials - even of the same species - as was shown by comparing the very uniform regenerants of the banana cultivar 'Prata Ana’ [139] with the highly variable regenerants of cultivar 'Valery’ [140]. When AFLP analysis was applied to regenerants of *Helichrysum italicum*, plantlets derived directly from leaves showed the same level of variability as plantlets that had passed through a callus stage [141]. Although only 6.2% of a total of 449 bands were polymorphic, almost all plantlets differed from the original genotype in at least one band. The same band polymorphism was encountered in several plantlets in some cases, suggesting a hot spot of DNA instability. In another study, plant material of date palm derived from asexual embryogenesis showed considerably more variability than plants derived from organogenesis when analyzed with AFLP markers [142].

Detailed sequence-based analysis of the molecular events responsible for SCAR marker polymorphism (for example, insertion or excision of transposons, microdeletion, recombination) between somaclones and sexual recombination-derived lines of maize, demonstrated that the same mechanisms apparently determine both *in vitro* and *in vivo* variability [143]. Therefore, it was concluded that cell culture only enhances the rate of heritable genomic changes which otherwise occur naturally in living organisms. Carrier and colleagues [144] studied somaclonal variation in the grapevine cultivar 'Pinot noir’ by high throughput sequencing and found that insertion polymorphism generated by transposable elements was responsible for most of the variation.

##### Forensic botany

In theory, DNA fingerprints obtained from plant fragments should be able to provide important evidence in crime investigations but success has been limited so far, probably due to problems with isolating DNA of sufficient quality from poorly preserved plant material. SSR markers are often chosen for forensic work since they work comparably well also with heavily degraded DNA. One famous early case, however, involved RAPD analysis of seed pods of the Palo Verde tree, *Cercidium* sp., recovered both from the crime site and from the pick-up truck of a suspect [145], while another case made use of SSR and RAPD analysis to compare fragments from clonally reproducing bryophytes (mosses) collected both on the crime site and on the suspect himself [146]. In subsequent experiments, a high likelihood of picking up fragments of bryophytes by walking outdoors wearing rubber boots was shown, as well as the ability to isolate DNA of sufficient quality after several months of storing bryophyte material under adverse conditions [147]. These facts together with the high level of clonality in many bryophyte species make them an ideal target for forensic analysis. In yet another criminal case, seedlings of the inbreeding herbaceous knotweed *Polygonum aviculare* obtained from germinating seeds found in the wheelhouse of a suspect’s car tire, and from a large number of soil samples taken at the crime site and various reference localities, were analyzed with AFLP [103].

Detection of adulterations of food, drink and medicinal products is another area for forensic botany. Licensing arrangements sometimes require that a specified clone, cultivar or landrace is utilized in the manufacturing of food and beverages. Thus, well-defined grapevine clones must be used to receive “appellation d'origine controllée” labelling in France. In one study, musts (that is, freshly pressed grape juice destined for wine-making) from two different grape cultivars could be identified using two SSR markers [148]. In another study, musts containing different proportions of two grape cultivars were analyzed with densitometry measurements of the SSR amplification products after separation and staining on polyacrylamide gels [149]. In Greece, Nemea wines are marketed with protected denomination of origin (PDO). Instead of using only the prescribed cultivar 'Agiorgitiko’ , the more productive 'Cabernet Sauvignon’ is sometimes added. DNA samples from fresh and fermented products, containing various mixtures of these two cultivars, were therefore subjected to a CAPS assay [150]. Presence of the adulterant could be detected down to 10% throughout the fermentation process.

Olive oil is also often marketed with PDO labelling. RAPD, ISSR and SSR analysis of Portuguese olive oils allowed the determination of geographic origin of the cultivars on which they had been based [151]. Similarly all 10 olive cultivars involved in samples of Italian oil samples could be identified with only one AFLP primer pair [152]. For rice, the adulteration of the expensive Basmati rice is an important issue, not only for European and US customs but also for consumers. Basmati cultivars have often been mixed with crossbred Basmati varieties and long-grain non-Basmati varieties. Several DNA-based markers have been proposed, and some were commercialized for adulteration tests, such as the multiplexed SSR markers developed by Archak and colleagues [153]. DNA analyses of various plant-based food products have similarly been used for authentication. The presence of the apple 'Annurca’ could thus be verified by SSR analysis in highly processed nectar and purée products [154]. Using relatively short SSR target sequences (below 160 bp), it was also possible to amplify genomic DNA from canned pear fruit and fruit juice while markers with longer target sequences failed [155].

Medicinal drugs constitute another important product area where adulterants cause major problems. Based on nine SNP sites, all populations except two could be distinguished in DNA isolated from the dried stems of the orchid *Dendrobium officinale*, which is a valuable source of 'Fengdou’ drugs used in traditional Chinese medicine [156]. The latter two populations could instead be distinguished using a more complex procedure known as suppression subtraction hybridization which involves PCR amplification, differential DNA fragment cloning and sequencing. Using these protocols, origination of the plant material could be determined for 50 drug samples obtained at a commercial market. For more information on DNA marker use in medicinal plants, see the reviews by Nybom and Weising [157] and Sarwat and colleagues [158].

A variety of DNA marker methods have been used to demonstrate infringement of Plant Breeder’s Rights, either in court or, in our experience much more common, leading to a settlement outside of court [159]. A related field concerns the identification of plants, the possession of which is considered illegal. Thus several studies have been published on the identification of *Cannabis sativa* specimens as part of drug enforcement [160]. In one approach, 15 SSR loci were combined into a single multiplex to enable fast and user-friendly discrimination between *Cannabis* genotypes [161]. One of the detected genotypes, however, proved to be very common in police seizure-derived evidence material, suggesting that many illicit growers had access to the same clone. This clonal propagation of course makes it difficult to determine the origination of a particular batch. A related DNA marker application concerns violation of trade restrictions. A special situation is encountered when products from protected trees are involved since woody tissue usually yields heavily degraded DNA. Nevertheless, a set of SNP markers derived from cpDNA intergenic spacers have proven useful for identification of tropical tree species using wood-derived DNA samples [162].

#### Genetic diversity, population structure and genetic relatedness

Discrimination among different genotypes is often only a starting point for the subsequent quantification of genetic variability among these genotypes and analysis of patterns of relatedness and gene flow. The extent of genetic variation in a species and its distribution among and within populations is determined by a large number of factors, such as the breeding system, historical events regarding, for example, habitat availability and immigration, population size, migration between populations and many biotic and abiotic ecological factors. Nybom and Bartish [163] compiled 106 RAPD-based studies and described the effects of several life history characters and sampling strategies on genetic diversity estimates. In another paper [96], 307 nuclear DNA marker studies (RAPD, AFLP and SSR) were compiled and investigated in a similar manner. One outcome of these surveys was that long-lived, outcrossing and late successional taxa retain most of their variation within populations, whereas annual, selfing and early successional taxa allocate more variation among populations. Within-population diversity is, in general, negatively correlated with the level of population differentiation.

The uniparentally inherited plastid genomes behave as a single, haploid character, and the effective population size for plastid markers is therefore only half of that of nuclear (diploid and biparentally inherited) markers. Consequently, population differentiation due to genetic drift occurs much faster for cpDNA markers than for nuclear markers. Because of their relatively high intra-specific variability, chloroplast and mitochondrial micro- and minisatellites are therefore very useful for studying genetic structure at a species-wide scale.

##### Population differentiation and gene flow

DNA markers have become a major tool for studying fundamental evolutionary influences of natural selection, mutation, gene flow and genetic drift on wild plant populations. While selection and colonization history is responsible mainly for large-scale structuring of genetic variation, gene flow and genetic drift operate also at a more narrow geographic scale. Among these factors, gene flow especially has received much attention since it is crucial in determining levels of species integrity and subdivision. As already mentioned, breeding system has a profound effect on gene flow and the partitioning of genetic variation between and within populations. The occurrence of IBD between populations has been demonstrated with DNA markers in many different kinds of outcrossing plant species such as, for example, the herb *Saxifraga oppositifolia*[164], the Brazilian peppertree *Schinus terebinthifolius*[165] and the Australian shrub *Grevillea mucronulata*[166]. IBD has been shown to occur, although much more seldom, also in selfing species such as wild emmer wheat, *Triticum dicoccoides*[167]. In accordance with these results, a correlation was found between collection distance and RAPD-based among-population diversity estimates for outcrossing taxa [163]. A corresponding association was, however, not found for selfing taxa.

In addition to the inherent dispersal capabilities of a species, gene flow is also affected by natural and anthropogenic habitat heterogeneity. Spatial autocorrelation analysis has thus become a valuable tool for studying spatial scale-dependent changes in DNA marker polymorphism within a population or group of closely occurring populations, and the impact of habitat characteristics on the resulting spatial genetic structure (SGS). Several computational methods have been used to calculate autocorrelation coefficients that measure the genetic similarity between individuals that fall within a defined distance class. A positive autocorrelation is frequently encountered over shorter distances, even if there is no overall linear correlation between geographic and genetic distances when calculated across the whole data set. Using RAPD data, Torres and colleagues [168] found significant autocorrelation in the first distance class (15 m) in populations of the endangered cliff specialist *Antirrhinum microphyllum*, suggesting a patchy distribution of genetic diversity. This is consistent with the territorial behavior of the main pollinator *Rhodanthidium sticticum*, short-distance seed dispersal, and a likewise patchy distribution of suitable habitats.

Many plant species comprise both central, so-called core populations as well as more or less peripheral populations. Such populations may experience considerable differences in the magnitude of operating evolutionary and ecological forces. For example, edge and core populations of the herb *Pulmonaria officinalis* exhibited strong differences in allelic and genotypic richness, expected heterozygosity and inbreeding coefficent when analyzed with SSR markers [169]. Similarly, an SSR analysis of eastern white cedar, *Thuja occidentalis*, showed that SGS could be detected over a six times larger distance (90 m) within peripheral populations compared to within core populations (15 m) [170].

Autocorrelation analysis has demonstrated IBD also in mainly selfing species but then usually at a very narrow scale, as was shown in the wild barley species *Hordeum spontaneum*[171].

Highly informative estimations of gene flow can be obtained by genotyping the same plant material using both nuclear and organellar markers. Since the former are biparentally inherited and the latter usually only maternally inherited, the resulting data provide an indication of the relative importance of pollen versus seed migration [172]. This ratio can vary by at least two orders of magnitude, and is typically much lower for insect- as compared to wind-pollinated plants [173]. In dioecious and therefore obligatory outcrossing plants, a mixture of autosomal and sex-linked SSR markers can provide direct evidence of the relative importance of seed versus pollen dispersal. Contrary to previous expectations, similar levels of pollen and seed dispersal were detected in the dioecius perennial plant *Silene latifolia*[174]. In selfing species, the lower incidence of inter-plant pollen transfer is expected to reduce the pollen to seed migration ratio, as verified by values well below unity at short distances within wild populations of *Hordeum spontaneum*[171].

While genes can move between populations by seed and/or pollen, colonization of new habitats is dependent on seed only. In coastal plants, seeds often have the potential to disperse over long distances by hydrochory. In a study of wild sea beet, *Beta vulgaris* subsp. *Maritima*, comprising more than a thousand plants from 33 populations along the French coast of the Anglo-Norman gulf, both mitochondrial and nuclear SSRs were applied [175]. Analysis of SGS and determination of zones of sharp genetic change demonstrated narrow IBD indicative of short-range dispersal, as well as genetic barriers fitting the orientation of marine currents and indicative of long-range seed dispersal.

Effects of an increased subdivision or fragmentation of natural plant habitats has received much attention lately; dispersal between populations is reduced as well as genetic diversity. Outcrossing species may especially suffer from enforced selfing or biparental inbreeding in fragmented habitats, and lose much of their potential for adaptation to changing environmental conditions. Using SSR markers, White and colleagues [176] compared fragmented versus continuous populations of the tropical tree *Swietenia humilis* in Honduras. Genetic variation was still high in all habitat fragments, but low-frequency alleles were more scarce, thus foreboding future genetic erosion. In another early study on tropical trees, Aldrich and Hamrick [177] reconstructed a population-level pedigree of *Symphonia globulifera*. Seedlings only occurred in primary and remnant forests, but not in pastures. Surprisingly, however, the majority of seedlings in fragmented forests proved to be derived from a few adult trees located in the open pasture land. Thus the genetic bottleneck experienced by the seedlings in remnant forest patches was caused by the reproductive dominance of a few spatially isolated trees in pasture land, in conjunction with unusually high levels of selfing in these trees.

Overall, tree species have been considered as comparatively resilient to fragmentation due to their often highly effective long-distance dispersal mechanisms. Recently, however, the wind-pollinated and wind-dispersed Andean tree *Polylepis multijuga* was analyzed with AFLP and shown to contain surprisingly little heterozygosity and to display SGS at short distances, suggesting that most seeds moved only a few meters [178]. This type of information is valuable when developing conservation plans for species protection and perhaps also for a possible reintroduction. Information about, for example, colonization and spreading behavior can be equally helpful when developing measures for stopping further growth of an invasive species. A combination of spatial genetic and geostatistical analyses of data from chloroplast and nuclear SSRs showed how the original two introductions of the invasive Brazilian peppertree *Schinus terebinthifolius* in western and eastern Florida, respectively, had spread and hybridized in little more than one century [165]. Since both long-distance jumps and short-distance diffusive spread could be demonstrated, highly concerted eradication efforts or the manufacturing of effective biocontrol agents are apparently called for.

##### Genetic relatedness

DNA fingerprinting data are often used to quantify levels of relatedness among genotypes or groups of genotypes, and numerous relatedness estimators have been described and compared. When wild plants are involved, the purpose is often to compare DNA marker-derived estimations of relatedness with current systematic treatment (see also "Applications of present-day DNA fingerprinting in plants" above). Other applications include parentage analysis, which is the most direct way to estimate gene flow. SSRs are the most commonly used markers for this purpose but data simulations have shown that multi-locus markers such as AFLP can also be used with high confidence, at least when the dominant alleles occur in frequencies of 0.1 to 0.4 [179]. Using SSR, a paternity analysis was conducted in a natural stand with two oak species, *Quercus robur* and *Q. petraea*[180]. The spatial distribution of male parents of the offspring from 13 maternal progeny arrays was determined, and the information used for calculation of pollen dispersal curves and analysis of gene flow. Similarly, gene flow was estimated from an SSR-based paternity analysis in the South American palm tree *Euterpe edulis*[181]. First, an exclusion analysis was performed by comparing adult and juvenile genotypes. After that, a paternity index was calculated among adults that could be the putative parents for a particular juvenile. Gene flow was shown to take place over longer distances than expected (up to 22 km), but it was not possible to distinguish between seed versus pollen transport. Since chloroplasts are paternally inherited in conifers, chloroplast simple sequence repeat (cpSSR) markers can, however, be very useful for direct estimates of paternity, as was demonstrated in white fir, *Abies alba*[182].

Access to correctly defined relationships can be very important in plant breeding for the calculation of heritability of specific traits. Various statistical formulae have therefore been developed for determining genetic relationships among individual plants. In a comparison of either purely marker-derived estimations of relationships or combined pedigree and marker-derived estimations, the latter proved to be more informative when analyzing Scots pine, *Pinus sylvestris*, offspring in a progeny test of open-pollinated genotypes in a seed orchard [183]. Surprisingly incongruent data were obtained when S-SAP markers, SNPs and pedigree data for a set of 35 wheat cultivars were compared [184]. The molecular methods produced similar estimates for the overall partitioning of genetic diversity between and within groups of cultivars, but the genetic similarities between pairs of cultivars were not correlated. SNP-based data were more closely associated with pedigree information than S-SAP-based estimates, probably because polymorphisms are strongly dependent on retrotransposon-related genomic rearrangements.

For cultivated crop plants, estimates of relatedness can provide valuable insights into the domestication process when material originating from different geographic areas is analyzed such as in, for example, Italian olive cultivars [121]. Relatedness among cultivated material on the one hand, and wild populations of the same or closely related species on the other hand, has also been addressed in, for example, apple using SSR, AFLP and cpDNA markers [185,186] (see also "Hybridization and introgression" below).

Estimating the true level of genetic relatedness among cultivars from nuclear DNA marker data is quite difficult, since the obtained information can usually only estimate identity by state (phenetic analysis) instead of the more desirable identity by descent (phylogenetic analysis). One interesting approach towards a true phylogenetic analysis has, however, been achieved within the HiDRAS project [187]. This project involves the analysis of specific chromosomal regions in genetically related apple cultivars using a large set of SSR markers that cover almost the whole genome. Thus, being able to accurately detect levels of genetic relatedness between different cultivars is very helpful for further analyses of, for example, QTL inheritance.

DNA marker-based procedures have frequently been applied to assess diversity and relatedness in collections of cultivated plant material - for example, gene banks. Interestingly, the anticipated loss of overall genetic diversity proved to be negligible when studied in 198 Nordic bread wheat landraces and cultivars that were developed during the last 100 years [100]. DNA markers are also highly useful for the purpose of setting up core collections within gene banks - that is, subsets of the entire plant material, chosen so as to preserve as much as possible of the initial diversity. Two main approaches have been used; the first with some kind of stratification using cluster analysis, and the second with methods for determination of genetic uniqueness. Numbers of retained SSR alleles can be maximized using a measure of uniqueness known as maximation strategy [188-190].

#### Genome constitution: hybridization, introgression and polyploidy

In contrast to animals, many plant groups are characterized by highly variable ploidy levels, often even within the same species. This addition of genomes has certain effects on DNA marker application and data treatment. Moreover, the formation of hybrids by fusion of gametes from two different entities (species, subspecies, and so forth) is also common in plants [191]. While homoploid hybridization takes place at the same ploidy level, most hybridization events instead involve the duplication of genomes, resulting in allopolyploid taxa.

##### Hybridization and introgression

In a series of classical studies, homoploid hybridization among American *Iris* species was investigated using a wide variety of DNA-based methods. First, *Iris fulva* and *I. hexagona* were shown to each have a species-specific rDNA profile [192]. Subsequently, DNA profiles indicated inter-specific hybridization as well as further introgression in both directions in populations where the two species co-occurred [193]. Diagnostic RAPD and cpDNA-CAPS markers were generated for these two species as well as for *I. brevicaulis*, and *I. nelsonii* was shown to have derived from hybridization between all three species [194].

Another important set of studies on homoploid hybridization has been undertaken in the sunflower genus, *Helianthus*. RAPD linkage maps were developed for the sympatric and hybridizing species *H. petiolaris* and *H. annuus* and subsequently used to analyze the genome of a recently formed hybrid species, *H. anomalus*, as well as of an artificially generated hybrid [191,195]. Later on, divergence between the two parental species was analyzed using 108 mapped SSR markers [196], and below average introgression was noted for SSR markers located close to QTLs for species differences when two parapatric species, *H. annuus* and *H. debilis*, were investigated [197,198]. Interestingly, gene flow was mainly in the direction from the hybrid back into these two parental species [199].

cpDNA-derived information has played a major role in elucidating many cases of homoploid hybridization and subsequent introgression. Studies of multiple taxa in several tree genera have thus shown that chloroplast haplotypes often are closer associated with geographic origin than with species affiliation - for example, in oak trees, *Quercus*[200], in *Eucalyptus* from Tasmania [201] and in the South East Asian pioneer tree genus *Macaranga*[202]. This introgression phenomenon has been coined “chloroplast capture” [203].

DNA markers are also commonly used for detecting both ancient and ongoing hybridization between crops and their wild relatives. *Malus sieversii* grows in Kazakhstan and has been suggested as progenitor of cultivated apple, *M. domestica*, based on morphological, historical and molecular evidence [204]. Nuclear SSR-based analyses have later been undertaken to investigate the genetic diversity and population structure in *M. sieversii*[205]. The origination of cultivated apple may, however, be more complicated. In another SSR-based study, three separate although partly overlapping gene pools were formed by (1) *M. sieversii*, (2) the European wild apple species *M. silvestris*, and (3) old and modern apple cultivars [186]. In the same plant material, analyses of chloroplast haplotypes produced rather unexpected results. Thus, *M. sylvestris* not only had the same common haplotypes as *M. domestica*, but there was also local sharing of uncommon haplotypes between the two species, suggesting recent inter-specific gene flow. A strong affinity between *M. sylvestris* and modern apple cultivars was likewise suggested in an SSR analysis of 839 genotypes collected from China to Spain, and representing four wild species as well as cultivated apple [206]. In this study, data were analyzed both with the computer program STRUCTURE, and with approximate Bayesian computation which offers a more historical perspective on gene flow.

Two variants of STRUCTURE, InStruct and NewHybrids, were used by Muranishi and colleagues [207] in a recent SSR-based study of *Magnolia stellata* and *M. salicifolia* together with putative F_1_ and F_2_ hybrids and backcrosses. The resulting clusters could be verified also with morphological trait analysis. Simultaneous application of cpDNA SSR markers showed that introgression was heavily asymmetric, with *M. salicifolia* being the seed parent of almost all hybrids and backcrosses.

In plant breeding, there can be good reasons for analyzing parental contributions in recently developed, experimental hybrids, especially if the breeding process has involved one or several generations of backcrossing. The amount of parental influence could thus be quantified using a microarray analysis with 7,680 probes simultaneously detecting SNPs, indels and methylation differences, in a set of intergeneric hybrids between the commercially important grass genera *Festuca* and *Lolium*[126]. The extent of similarity between the derived *Festulolium* cultivars and the parental genomes was clearly associated with the type of crossings performed - that is, F_1_, F_2_ or backcrosses.

##### Polyploidy

Polyploidy is very common in the plant kingdom. Although the same marker technologies can be used for genotyping diploid as well as polyploid samples, statistical analyses and interpretations are usually less straightforward when polyploid samples are involved. Many species are allopolyploids and have been derived from their diploid ancestors by hybridization. Moreover, molecular studies of allopolyploid taxa and their putative progenitor taxa have shown that multiple origination is the rule rather than the exception. While frequent gene flow between polyploid lineages and back-crossing to parental taxa can further confound this process, a more easily studied case is offered in apomictic species where the speciation event is more or less frozen in time. One such example is the North American allopolyploid cloak fern, *Astrolepis integerrima*, which was recently studied by cpDNA sequencing and AFLP analysis [208]. Six relatively localized cpDNA haplotypes were detected, some of which were further divided by AFLP. All in all, the results suggested that a total of 10 *A. integerrima* lineages have been formed through multiple independent hybridizations between *A. cochisensis* and *A. obscura*.

Identification of the putative progenitor species of polyploids can be attempted with various types of markers, including the internal transcribed spacer (ITS) region in the nuclear ribosomal RNA gene clusters which was sequenced and analyzed in, for example, polyploid rose cultivars and species [209]. Nair and colleagues [210] used IRAP primers to determine the genomic constitution in a set of mostly triploid banana cultivars. Primer sequences were derived from two different retrotransposons, one occurring in the A genome (*Musa acuminata*) and the other in the B genome (*M. balbisiana*). A more easily applied CAPS marker, obtained from PCR amplification of the ITS region followed by restriction with *Rsa*I, has also been applied for this task [211]. More recently, application of 653 DArT markers similarly allowed the discrimination between A and B genomes, and the identification of these genomes within a set of banana cultivars [212].

Multi-locus based methods such as RAPD and AFLP are sometimes used for studying population genetics in species with different ploidy levels, but problems can arise due to a positive correlation between ploidy level and number of scored bands [213]. In addition, banding patterns may differ qualitatively between samples at different ploidy levels, and thus give rise to scoring errors. Single-locus markers such as SSRs and SNPs are also problematic due to the occurrence of multiple alleles and complex segregation ratios. SSR markers may be more or less genome (and species)-specific and therefore fit only one of the two homologous genomes of an allopolyploid hybrid, producing no amplification in the other (null alleles, or allele drop-out). Truly genome-specific SSR loci that consistently produce a maximum of only two alleles in each sample are rare, but can be quite useful as demonstrated in the hexaploid *Mercurialis annua*[214]. With these markers, population genetics parameters could be calculated as if the species instead were diploid.

For allopolyploid crops with intermediate levels of similarity among homologous genomes, such as tetraploid potato, SSR primers in general produce a variable number of bands per locus. For example, Fu and colleagues [215] found a total of 64 alleles when investigating 169 potato accessions with 36 SSR primer pairs. Even apparently diploid species such as apple may be “ancient polyploids” in which some primer pairs can produce a second set of alleles derived from an unrecognized duplicated genomic area [187]. Amplification of these supernumerary loci (isoloci) frequently varies with the experimental conditions, and can cause problems when data are being combined from several laboratories.

In autopolyploids and in allopolyploids with low genomic differentiation, SSR analyses usually produce multiple alleles of a single locus in each genotype, as demonstrated in the autopolyploid and apomictic *Ranunculus kuepferi*[216] and in allopolyploid species and cultivars in the genus *Rosa*[217-219]. To fully utilize the information content of the obtained DNA profiles, segregation patterns must be determined. This, however, requires the ability to score allele dosage, in contrast to just the presence or absence of an allele. The MAC-PR approach (microsatellite DNA allele counting - peak ratios) determines allele copy number based on quantitative differences between microsatellite allele peak ratios and therefore allows the precise determination of allelic configuration in each studied sample, as was shown in tetraploid roses [217,220]. Using this approach, inheritance patterns have been studied even in the absence of experimental crosses [221]. High-quality banding patterns are, however, needed for successful application of the MAC-PR, as well as repeatability of relative allelic amplification intensities among individuals and, thus, homology of microsatellite marker alleles within a species.

In crop plants with detailed pedigree information, the so-called microsatellite allele dose and configuration establishment (MADCE) procedure can be used to trace the transmittal of SSR alleles through documented generations of the investigated plant material, and determine the exact allele copy number in the target cultivars [222]. Originally, the MADCE procedure was applied in apple [222] but informative results have recently been obtained also for the Strawberry Crop Reference Set within the RosBreed research project (Bassil N, personal communication).

In many situations, allele dosage can, however, not be accurately scored with the methods chosen, and much plant material lacks or has only unsatisfactory pedigree information. Specialized programs have therefore been developed for analyzing polyploids with SSR markers, such as, for example, TETRA [223] and POLYSAT [224]. The fitTetra R package has been developed for enabling genotype calling in tetraploid species from biallelic marker data, and is especially useful for large-scale SNP analyses in material with high levels of polysomic allele segregation such as potato [225]. By contrast, the bead array MSV package [226] appears to be more useful for material with mainly disomic segregation.

A major drawback with any multi-locus approach is the loss of information about exact levels of heterozygosity and about genome inheritance. In addition, genetic distances between cultivars are exaggerated as compared to distances calculated on the basis of co-dominant data [220]. A method for calculating genetic distances that permits unbiased comparisons between different ploidy levels has, however, been described [227] and is available in the computer program package GENOTYPE/GENODIVE. Another approach is based on the formation of multi-locus allele phenotypes of each investigated individual, and calculation of phenotype-based estimates of genetic diversity and differentiation [228]. For more information on methods to describe the population genetics of polyploids, see Assoumane and colleagues [229].

#### Plant speciation, phylogeny and systematics

As the availability of DNA-based information increases, more attention is being paid to the genomic patterns of differentiation among plant species. According to the genic view of plant speciation, small “genomic islands” may be responsible for much of the differentiation between taxa through divergent selection or reproductive isolation barriers, while the remainder so-called “porous genome” is more permeable to gene flow [230,231]. In this context, the choice of molecular method becomes crucial for the ability to reflect genomic differentiation in a phylogenetically relevant perspective. To determine relative marker sensitivity in monitoring inter-specific differentiation, Scotti-Saintagne and colleagues [232] conducted a genome scanning experiment with 389 markers (allozymes, AFLPs, SCARs, SSRs and SNPs) on samples from pairs of populations of the sympatric oak species *Quercus robur* and *Q. petraea*. Distribution of markers according to their ability to detect inter-species diversity was clearly L-shaped; apparently only a few markers were located in genomic regions responsible for species differentiation. As expected, these markers were more likely to reside in coding regions than in non-coding regions. In another genome scan based on 88 mapped SSR loci, most loci again showed considerable migration between the analyzed taxa: sunflower species *Helianthus annuus*, *H. debilis* and their inter-specific hybrid [199]. The genomic regions that are responsible for genetic differentiation therefore appear to be small in these taxa, whether estimated as level of species differentiation or as migration rates.

When targeting differentiation at a larger taxonomic scale, DNA sequence information of the chloroplast genome and/or nuclear genic regions, is usually preferred over DNA fingerprinting. Proper elucidation of the complex puzzle of plant systematics is, however, often best achieved with a combination of different types of molecular information. In many plant groups, various types of multi-locus or single-locus markers have thus provided important pieces to the puzzle. So far, these marker loci have seldom been placed on a genomic map (but see, for example, [199,232]), and whether they reside in a conserved versus a “porous” part of the genome is usually unknown. Instead, choice of markers has mainly been based on the feasibility of obtaining a sufficiently large number of polymorphic bands.

Multi-locus DNA profiling methods such as AFLPs have become the most commonly used DNA fingerprinting tool in plant systematics, mainly in situations where DNA sequencing produces insufficient phylogenetic resolution [233]. In an early plant systematic study using AFLPs, 551 polymorphic bands were obtained with three primer combinations for 30 accessions from 19 taxa of *Solanum* section *Petota* and three taxa of *Solanum* section *Lycopersicum*[213]. Ploidy level was reflected in the profiles, with hexaploids exhibiting more bands than tetraploids and diploids. Mating system had, as expected, a large impact, with 40 to 60% intra-specific polymorphism detected in outcrossing taxa as compared to only 0 to 2% in selfing taxa. AFLP methodology was also employed to investigate phylogenetic relationships among 43 species of the paleotropic pioneer tree genus *Macaranga*[234]. About 30 of these species have a symbiotic relationship with specific ant partners. The resulting phenograms supported the monophyly of several sections and subsectional groups within the genus, and provided evidence for a polyphyletic origin of the ant-plant mutualism.

Besides the two species of cultivated rice, the genus *Oryza* also comprises of around 22 wild species that have received considerable attention due to their potential importance for rice breeding. Six diploid genomes (A, B, C, E, F and G) and four allotetraploids (BC, CD, HJ and HK) have been identified using, among other methods, total genomic DNA hybridization [235]. In another early study on rice, 77 samples representing 23 *Oryza* species were analyzed with AFLP [236]. Pairwise genetic distances showed a linear increase depending on the taxonomic level, with 0.02 to 0.21 within species, 0.2 to 0.35 between species sharing the same genome type, and >0.7 between species carrying different genomes. For the subsequent analysis of phylogenetic relationships among these genomes, more conserved markers were developed through the identification and sequencing of numerous rice genes [237]. Comparison of sequences for 142 such genes in six species, representing the six different diploid genomes, allowed the reconstruction of the rapid diversification in *Oryza*. In a follow-up study based on the sequences of 106 nuclear genes, divergence times and ancestral effective population sizes were also determined [238].

In the large and complex genus *Rosa*, several different DNA-based methods have been applied for phenetic and phylogenetic analyses, with mostly consistent results - for example SSR [239] and AFLP [240]. Two major clades were identified, with sections *Carolinae*, *Cinnamomeae* and parts of *Pimpinellifoliae* forming one clade and most of the other seven commonly recognized sections forming the other clade. As for the division into sections, *Synstylae* appears to be mainly monophyletic and rather closely affiliated with sections *Indicae* and *Rosa*[240]. Furthermore, section *Pimpinellifoliae* is apparently polyphyletic, and *R. spinosissima* should be separated from the other species in this section. In spite of its size (currently, about 50 species are acknowledged) and hybridogenous origination, the mainly European section *Caninae* (also known as dog roses) apparently constitutes a well-circumscribed monophyletic group. Another, very large AFLP study was recently conducted on >900 dog rose specimens sampled in a transect across Europe, with more than 200 non-dog rose samples analyzed for comparison [241]. Two lines of statistical analyses were applied: (1) an unstructured model with principal coordinate analysis and hierarchical clustering, and (2) a model with a superimposed taxonomic structure based on analysis of genetic diversity using a novel approach that combines assignment tests with canonical discriminant analysis. Support was found for five of the seven subsections, including the three major ones: *Caninae*, *Rubigineae* and *Vestitae*. Within the subsections, many species overlapped considerably, and geographic distances often appeared to be at least as important as the conventional taxonomy in explaining similarities between analyzed specimens. Complementary information on phylogeny in *Rosa* has also been obtained with DNA sequencing. Although sharing some ITS sequence types with species in other sections thereby confirming their hybridogenous origin, the *Caninae* species also have one unique ITS sequence type which is further evidence of their monophyly [209,242].

Plant systematic studies have occasionally been conducted also using SSR markers, especially when the focus has been on genetic differentiation among closely related taxa. For example, ten Puerto Rican populations of the cycad genus *Zamia* were analyzed with 31 SSR primer pairs [243]. These populations could be treated either as belonging to a single polymorphic species, *Z. pumila*, or as representing three more narrowly circumscribed taxa: *Z. erosa*, *Z. portoricensis* and *Z. pumila sensu stricto*. The SSR analysis showed that *Z. erosa* is strongly differentiated from the other two species, and thus may represent an independent introduction into Puerto Rico. The data are consistent with an allopatric speciation scenario with *Z. portoricensis* being the youngest taxon according to Bayesian coalescent analysis and effective population size, and still showing considerable admixture with *Z. pumila*.

Genetic relationships among 35 *Arachis* species from seven sections, including 11 accessions of cultivated peanut, *A. hypogaea*, were analyzed on the basis of allelic variation at 32 SSR loci [244]. A neighbor joining tree was generated on the basis of pairwise Dice distances between individual accessions, calculated from a binary presence/absence matrix of SSR alleles. Most con-specific accessions grouped together on the tree, as did species from the same section, with several exceptions that were attributed by the authors to either homoplasy in the dataset or extensive within-species variation.

From the relatively few studies available, it appears that SSR markers do have some potential not only for species delimitation, but also for the reconstruction of genetic relationships among closely related species groups that are only a few million years old. However, SSR markers are usually highly polymorphic and therefore multiallelic within a species. Accordingly, the within-population component of SSR variation is often much higher than the between-population or between-species component [245]. It is therefore a “must” that several accessions per species are included in any phylogenetic study that is based on SSRs, the more the better. Optimally, genetic distances between populations (or species) rather than genetic distances between individuals should be used to generate phenetic trees.

#### Phylogeography

Phylogeography aims to study the spatio-temporal history of a species on the basis of its intra-specific genetic variation [246]. In principle, phylogeographic studies can be based on information from either nuclear, mitochondrial or chloroplast DNA. In practice, organellar DNA is usually preferred since organelle-derived markers are more likely to retain information about biogeographical history than nuclear markers [247]. There are several reasons for this. First, the haploid genomes of plastids and mitochondria exhibit a smaller effective population size as compared with the diploid nuclear genome, resulting in stronger substructuring of fragmented populations under genetic drift. Second, organellar genomes are usually inherited uniparentally. In angiosperms, the plastid DNA is generally transmitted by seeds - that is, maternally. Given that plants can colonize a new habitat only by seeds, plastid-derived markers have the potential to provide information about past changes in species distribution that is unaffected by pollen flow. Third, intermolecular recombination is usually absent in plastid DNA, so that individual sequence polymorphisms can be combined into haplotypes that remain mostly unchanged when passed to the next generation.

Evolutionary relationships between cpDNA haplotypes are often depicted as networks [248], which can be superimposed on the geographic distribution of the sampled plants. One has to keep in mind, however, that a non-recombining DNA molecule behaves like a single gene. The phylogeographic pattern retrieved from a plastid haplotype network therefore only represents one out of several possible outcomes of the genealogical process [249]. This is why phylogeographic analyses based on other genes and genomes are becoming increasingly popular. In conifers, where plastid DNA (paternal) and mitochondrial DNA (maternal) show contrasting modes of transmission from parents to offspring, both genomes have often been analyzed side-by-side [250,251]. In addition, phylogeographic studies often employ nuclear ribosomal ITS sequences. In most plant species, the ribosomal genes are rapidly homogenized by concerted evolution and then behave like uniparentally inherited organellar DNA.

There is no clear division between phylogeography on the one hand and traditional population genetics on the other. Accordingly, the use of nuclear SSR markers to study genetic diversity, genetic subdivision and gene flow within and among extant species is sometimes also called “phylogeography” [252], and there have been numerous successful attempts to elucidate intra-specific phylogeographic patterns by multi-locus DNA profiling methods such as RAPD, ISSR and AFLP [253,254]. Multi-locus banding patterns are typically analyzed phenetically - that is, phenograms or networks are reconstructed on the basis of a pair-wise similarity matrix that is generated from a binary presence/absence matrix of band positions. The (groups of) genotypes depicted in the resulting phenogram or network are then compared with their geographic distribution [253].

The majority of plant phylogeographic studies still rely on plastid DNA polymorphisms that can be searched for by either PCR-RFLP, screening of length-variable plastid microsatellites (cpSSRs), or by comparative sequencing of PCR-amplified non-coding DNA [255]. Unique polymorphisms are then combined into distinct haplotypes, followed by the analysis of haplotype distribution and frequencies in different geographical regions, quantification of the genetic divergence between haplotypes, and the evaluation of genetic relationships between haplotypes - for example, in the form of a statistical parsimony network such as TCS [256]. The use of cpSSRs is, however, controversial, since their often high mutation rates can cause homoplasy [257,258].

Application areas of marker-based phylogeographic studies are diverse, and include, for example, the analysis of postglacial re-colonization patterns of the Central European landscape by trees and shrubs in the Quaternary [252,259], the identification of glacial refugia [260], the reconstruction of migration routes of halophytes along coastal and inland salty habitats [253], the investigation of the evolutionary history of tropical trees [255] and the historical biogeography of threatened species [251]. Increasingly important are comparative phylogeographies that involve numerous animals and plants from the same geographical region [261,262]. Such meta-analyses yield invaluable data on common evolutionary patterns across many biota from large geographical areas.

#### Genetic mapping

##### Linkage mapping and genetic maps

One prominent application of molecular markers is the generation of genetic maps which have been established for all major and many minor crops and other plants (for example, rice [263], barley [264], and maize [265], to name just a few). A genetic map is a graphic representation of a chromosome (or linkage group) onto which genetic elements (= loci, for example markers or genes) are aligned. The loci are arranged based on their co-segregation during meiosis, which depends on the frequency of recombination events. Genetic distances between loci are measured in centiMorgan (cM). One cM is defined as the distance that two loci have to each other, if in 100 meiotic events the loci are segregating only once (= 99% chance of co-segregation). As the extent of recombination varies in different genomes, this translates into varying physical distances. The recombination frequency also varies among different genomic regions - for example, recombination is suppressed near centromeres.

To estimate genetic distances among loci, the co-segregation of genetic elements is monitored in mapping populations or in association mapping approaches (see below). Mapping populations usually originate from a cross between two parental lines, which ideally can be distinguished by a large number of polymorphisms that are monitored in the progeny. Particularly convenient mapping populations consist of so-called Recombinant Inbred Lines (RILs), which are generated by selfing single-seed descent from different sibling F_2_ plants through six or more generations. The continuous selfing causes very high levels of homozygosity, and each RIL from a population of RILs hence conserves one particular recombinational event from the F1 cross. Design and construction of RILs have been reviewed by Pollard [266].

To identify loci that are very tightly linked with a specific trait, “fine-mapping” is performed by enriching the density of markers in proximity of the responsible genes or, in the best case, markers for the responsible genetic elements themselves. The most commonly applied technique for fine-mapping is Bulked Segregant Analysis (BSA), originally developed by Michelmore and colleagues [267]. In a BSA, all genotypes that show a specific phenotype (that is, a specific trait) are pooled and screened for polymorphisms that distinguish them from the remaining plants. All genetic elements that do not influence the bulk-trait are randomly distributed among all plants, whereas all genetic elements responsible for the trait are to be found preferentially if not only in the respective bulk. In consequence, any difference between the bulk and the remaining plants is likely to be linked with the trait of interest. The source of polymorphisms can be, for example, the metabolome, the proteome, the transcriptome or the genome. The latter two have profited enormously from the advent of high-throughput-sequencing technologies and are now the most widely used sources for genetic polymorphisms.

Different types of markers can be combined into integrated maps, which become more highly resolved (that is, saturated) with each newly added marker. Furthermore, data from different crosses can be integrated in the same map. For example, Wenzl and colleagues [264] published an integrated map for barley using DArT, SSR, RFLP and STS markers, altogether comprising 2,935 different loci. In the current era of genome sequencing, genetic maps are also a versatile tool for defining the order of assembled contigs from shotgun sequencing approaches, as has been done, for example, during the assembly of the recently published chickpea genome [268].

##### Association mapping

Association mapping (AM) aims at linking phenotypes to genotypes, independent of the kinship of the genotypes. The concept of AM has been implemented in humans and model organisms for many years (for example within the human HapMap project that started in 2002), and is now increasingly applied for plant genomes (see the reviews by Abdurakhmonov and Abdukarimov [269] and Soto-Cerda and Cloutier [270]). The major advantage of AM over linkage mapping (LM) or QTL mapping is that no mapping population is required. The establishment of good mapping populations is a time-consuming and costly task, especially for plants with long generation times and hence a limited number of meiotic recombinations. Furthermore, LM is usually restricted to a small subset of genotypes and to those loci that are polymorphic among these genotypes (= low allelic richness). In contrast, AM examines genotype-phenotype correlations in a large germplasm and hence monitors the historical meiotic recombination events that accumulated in natural populations and collections of landraces, breeding materials and varieties [270].

Association mapping is based on the occurrence of Linkage Disequilibrium (LD) between a particular trait and one or more alleles of a marker locus in a population. In contrast to LM, which refers to the combined inheritance of loci due to their close physical proximity on the same chromosome, LD refers to the non-random occurrence of allele combinations of loci in a population. Thus, the reason for LD can be linkage (and in most cases it is), but also other factors influence LD, such as selection, mutation, mating system, population structure, and so forth, which can result in significant LD even of alleles that are located on different chromosomes [270]. Because of this, AM is more complex than linkage mapping and might be biased by various factors.

More recently, AM-based analyses have been successfully carried out in many crops. In rice, for example, Zhao and colleagues [271] genotyped more than 44,000 SNPs across 413 accessions from 82 countries. Dozens of variants could be identified that influence numerous complex traits. In maize, a high-density analysis based on 56,110 SNPs was performed to analyze chilling tolerance in 375 inbred lines [272]. Nineteen highly significant association signals that explained between 5.7 and 52.5% of the phenotypic variance observed for early growth and chlorophyll fluorescence parameters were identified. An AM-based approach that was termed “landscape genomics” aims at simultaneously examining the effects of demographic history, migration and selection in a defined geographical site (see, for example, Sork and colleagues and references cited therein [273]).

Thanks to new, high-density genotyping methods such as the INfinumHD assay that assesses thousands of markers simultaneously, almost all major crops can now be subjected to AM [91]. High-density SNP arrays that comprise the information of several thousand loci were recently also developed for forest trees and horticultural plants, including white spruce, *Picea glauca*[274], peach [275], apple [276] and sweet and sour cherry [277], and are expected to greatly facilitate AM also in these plant species. A combination of LM and AM represents a particularly powerful tool for selection. Thus, Yu and colleagues [278] presented a so-called “nested association mapping” (NAS) approach in maize that involved crossing of 25 different variants and 5,000 offspring, whereas Kover and colleagues [279] performed a “Multiparent Advanced Generation Inter-Cross” in *Arabidopsis*. Nineteen variants were crossed in a random mating scheme, resulting in 527 F4 plants. The RILs originating from the maize NAS recently helped to identify important genes involved in maize kernel composition [280], resistance to northern leaf blight [281] and stalk strength [282]. In apple, Khan and colleagues [283] used an AM and LM combined approach to identify three important QTLs for fire blight resistance.

##### Marker-assisted breeding and genome-wide selection

One major aim of genetic linkage analysis in crop plants is marker-assisted breeding (see the review by Jiang [284]). A particularly promising current concept of marker-assisted breeding has been termed “genomic selection” (GS) or “genome-wide selection” (GWS) [285]. In contrast to the traditional marker assisted selection (MAS) concept, where only a subset of markers is considered, in GS all available markers are evaluated simultaneously for the calculation of a so-called breeding value. This is done by combining major and minor QTLs according to Meuwissen and colleagues [286]. In this way, QTLs with only minor positive and negative effects that are missed in traditional MAS are also taken into consideration for selection. The concept of GWS is widely used in livestock breeding and has been discussed as a future selection approach also for plants [287]. In their analysis that was based on a large data set of 25 nested association mapping populations, Guo and colleagues [285] found better predictions using the GWS approach as compared with MAS for flowering traits in maize (days to silking, days to anthesis and anthesis-silking interval). MAS was performed by composite interval mapping (see the review by Zou and Zeng [288]), and GS using a “ridge regression-best linear unbiased prediction” to calculate breeding values.

For genotyping moderate numbers of SNP loci in hundreds to thousands of samples, PCR-based approaches provide a more flexible alternative to microarray-based methods. Besides direct sequencing, three currently popular methods for SNP genotyping of PCR products are high-resolution melting (HRM) analysis, allele-specific PCR (ASP), and the TaqMan assay. In HRM analysis, the PCR product is continuously heated, and the separation of the two DNA strands is monitored in real time [289]. Polymorphic PCR products that differ slightly in length or sequence will have different melting temperatures. These differences can be measured with sensitive optics, which monitor the fluorescence-to-signal intensity of an intercalating dye. HRM analysis has, for example, been successfully applied to gene mapping in rice [290], to cultivar identification in sweet cherry [291], and to discriminate between closely related chloroplast DNA haplotypes in the wild species *Arenaria ciliata* and *A. norvegica*[292].

In ASP, the alternative alleles at a particular polymorphic site are amplified with allele-specific primers that are each labelled with a different fluorochrome. The presence of a particular allele is hence indicated by a diagnostic fluorochrome signal. If primers of different length are used, the ASP products can also be assessed by gel electrophoresis. The TaqMan™ assay dates back to the early 1990s [293]. It involves the fluorescence-based detection and quantification of a specific probe that is hybridized to the SNP site of interest. A light signal is only emitted when the probe is degraded by the exonuclease activity of the *Taq* DNA polymerase, which occurs only when the probe has specifically bound to its target site. The ASP and TaqMan™ assays can be assessed in regular quantitative PCR machines while HRM requires specific optics.

#### The status of traditional DNA fingerprinting: concluding remarks

Taken together, the last two decades have witnessed a prominent increase in the application of various DNA markers for plant DNA fingerprinting. In the beginning, multi-locus dominant markers, especially RFLP, RAPD, AFLP and ISSR, were most popular, but single-locus SSRs and eventually SNP markers rapidly caught up. The chip-based DArT technology is also still used. We believe that traditional multi-locus methods and their various spin-offs will still be employed a decade from now, but mostly for exploratory research that does not necessarily result in published papers. Locus-specific SSR markers will probably remain more popular, due to their co-dominant inheritance, ease of analysis and the fact that new data can easily be added to already existing files. Novel input to DNA fingerprinting was, however, provided by an unforeseen major breakthrough in DNA sequencing technology, which can be envisaged as the starting point for the future of DNA fingerprinting, and which is discussed in the following section.

## The future of DNA fingerprinting

The advent of massively parallel high-throughput genomic sequencing about 10 years ago was a breath-taking step forward in the yet short history of molecular genetics and genomics (see, for example, the reviews by Mardis [294], Metzker [295], and Rothberg and Leamon [296]). The immense speed with which DNA sequences can be read by 454 pyrosequencing, SOLID, Illumina and other machines also had a considerable and two-fold impact on the development and use of molecular markers in general, and on DNA fingerprinting in particular. On the one hand, conventional markers such as microsatellites and SNPs can now be discovered with reduced cost and effort and at unprecedented rates. On the other hand, DNA marker technologies are currently successively complemented or even replaced by the sequencing process itself, as is expressed by the term “genotyping-by-sequencing” [87,297,298] (see also the Special Issue of *Molecular Ecology* 22(11), 2013).

### Discovery of nuclear microsatellites by high-throughput DNA sequencing

Quite obviously, random high-throughput sequencing of genomic DNA offers itself as a useful strategy to identify all kinds of repetitive DNA in a genome, including microsatellites. It nevertheless took several years before this potential was realized. In one of the first reports on the use of next-generation DNA sequencing for microsatellite marker development, Abdelkrim and colleagues [299] used a 454 platform to produce 17,215 reads of unselected, fragmented genomic DNA of the blue duck (*Hymenolaimos malacorhynchos*), a waterfowl species endemic to New Zealand. Each read had an average size of 243 bp, adding up to a total of approximately 4.1 Mb. Using appropriate bioinformatic tools, microsatellites were detected in 231 reads. The number of suitable marker loci was, however, reduced to 24 by the necessity to design primers on either side of the microsatellite. Thirteen of the primer pairs displayed polymorphism, and 13 markers were thus generated in a single sequencing run.

Santana and colleagues [300] also used 454 technology to create microsatellite markers from a fungus (the pine pathogen *Fusarium circinatum*), an insect (the wasp *Sirex noctilio*) and a nematode (*Deladenus siridicola*). Two methods, ISSR-PCR and “fast isolation by AFLP of sequences containing repeats” (FIASCO), were used to enrich the template DNA for microsatellites prior to sequencing. Altogether, 1.2 to 1.7 Mb of DNA were sequenced, and 873 potentially amplifiable microsatellites were identified with sufficient flanking sequence available for primer design. A set of 28 SSR-flanking primer pairs were developed for *Fusarium circinatum*. Of these, 19 yielded single fragments in the expected size range, and 13 produced polymorphic amplicons from a set of fungal isolates. The authors also generated a traditional library from *F. circinatum* DNA enriched for microsatellites by the ISSR method. Sanger sequencing of 100 clones from this library yielded only eight potentially amplifiable microsatellites.

Again using a 454 platform, Allentoft and colleagues [301] searched for microsatellites in an ancient DNA source, a bone fragment of an extinct New Zealand moa species (a flightless bird). A total of 79,796 sequences were obtained with an average length of 112 bp. Of 195 di-, tri- and tetranucleotide repeats present in the data set, only one polymorphic microsatellite marker could eventually be generated. This low yield can be accounted for by the ancient source and therefore degraded state of the DNA and hence short read lengths.

The above three papers were published in the same issue of *BioTechniques* and indicated for the first time that high-throughput sequencing has a strong potential to isolate SSR markers from non-model organisms where no genomic information exists. The novel methods save a lot of time, and are also cost-efficient as compared to traditional enrichment cloning. For example, Csencsics and colleagues [302] spent approximately US$5,000 to develop microsatellite markers for the plant species *Typha minima* within 6 weeks. In their study, 307 di-, tri- and tetranucleotide repeats were found in a total of 76,692 sequence reads. One hundred loci were selected for primer design, 30 primer pairs were tested and yielded 17 polymorphic markers.

The relatively long read-lengths provided by 454 sequencing facilitate the identification of enough flanking sequence on either side of the SSR for the design of PCR primers. 454 technology was therefore initially preferred over other next-generation sequencing approaches for generating SSR markers. In plants, 454 sequencing was first applied to microsatellite isolation in *Typha minima*[302], *Amaranthus tuberculatus*[303] and *Vigna radiata*[304]. Many more studies followed, and genomic shotgun sequencing soon replaced traditional enrichment strategies as the method of choice for generating sets of microsatellite markers in any organism (see the review by Zalapa and colleagues [305]). More recently, SSR identification from paired-end genomic sequencing using the more economical Illumina platforms has been advocated by several working groups [306,307], especially since average lengths of Illumina reads have considerably increased over the years. For example, Castoe and colleagues [306] showed in one snake and two bird species that 454 and Illumina detect similar numbers of potentially amplifiable SSRs on a read-by-read basis, but the much lower costs per sequenced nucleotide are clearly favoring Illumina.

Given that sequencing methodology is still developing at a rapid pace, a further decrease of costs may be expected. The large amount of sequence data obtained in even a small-scale experiment allows a top-down selection of the most promising candidates (for example, only trinucleotide repeats, or only perfect repeats). Enrichment for microsatellite motifs prior to sequencing is sometimes advocated [307,308], but is probably unnecessary in most cases [306]. The use of (bar)coded adapters and primers for the amplification step enables the parallel sequencing of multiple templates at the same time [307,309], which further contributes to the efficiency of the method. Using barcoded adaptors (also known as multiplex identifier adaptors), Takayama and colleagues [310] were thus able to isolate a large number of microsatellites from only 10,000 to 20,000 genomic 454 reads each of six unrelated plant species at low cost.

Concerning the read-length of high-throughput sequencing platforms, a major breakthrough was recently reached by Pacific Bioscience, whose SMRT cells produce read-lengths of up to 15 kb. Sequence quality is, however, relatively low. It was therefore suggested to additionally produce reads with another sequencing platform (for example, Illumina) to correct the long PacBio reads in a hybrid sequencing approach [311]. Another alternative relies on the circularization of template molecules prior to single-molecule sequencing, and multipass sequencing of the same circular templates was reported to generate highly accurate consensus sequences [312]. Most recently, Grohme and colleagues [313] demonstrated for the first time the successful use of single-molecule circular consensus sequencing on a PacBio RS platform for discovering SSR markers in the genome of a goose, *Anser albifrons*. Whatever technology is used for sequencing, it is essential that appropriate bioinformatic tools and hardware are available to cope with the huge numbers of sequences that have to be screened.

An additional benefit of the use of next-generation sequencing for SSR marker development is the large amount of random nuclear and organellar sequence data generated as a “by-catch” of microsatellite identification. For example, 382 SSRs were sampled in the course of 454 sequencing of *Amaranthus tuberculatus* along with a contig representing an almost complete chloroplast genome, mitochondrial DNA fragments, transposable elements and numerous nuclear genes of interest [303]. Krapp and colleagues [314] assembled approximately 84% of the *Dyckia marnier*-*lapostollei* (Bromeliaceae) chloroplast genome from only 59,624 pyrosequencing reads. A total of 34 chloroplast microsatellites (cpSSRs) with a minimum number of 10 A´s or T´s were also found, and flanking primers were constructed that produced highly polymorphic amplification products in various *Dyckia* species.

### High-throughput genotyping-by-sequencing

The radically increased throughput in sequencing capacity at 50 to 100,000 times lower costs per base as compared with traditional Sanger sequencing also had a large impact on DNA fingerprinting technology itself. High-throughput sequencing opened completely new and unprecedently fast avenues for genotyping (up to the highest possible resolution) the resequencing of the entire genomes of populations of plants (WGS, for example [315,316]). Whereas the list of fully sequenced plant genomes is constantly growing (for crop plants, see Bevan and Uauy [317]), most plant genomes are nevertheless too large, too complex, and too rich in repetitive DNA to be good candidates for WGS. This is why essentially all genotype-by-sequencing approaches aim at reducing the complexity of genomes to a smaller subset (“reduced representation sequencing”; for example, see [298,318-324]). By reducing the analyzed genome space to a manageable size, a sufficiently high sequence coverage can be attained that allows for the detection of polymorphisms (mostly SNPs) with necessary confidence, even in plants with very large genomes.

**Figure 4:**
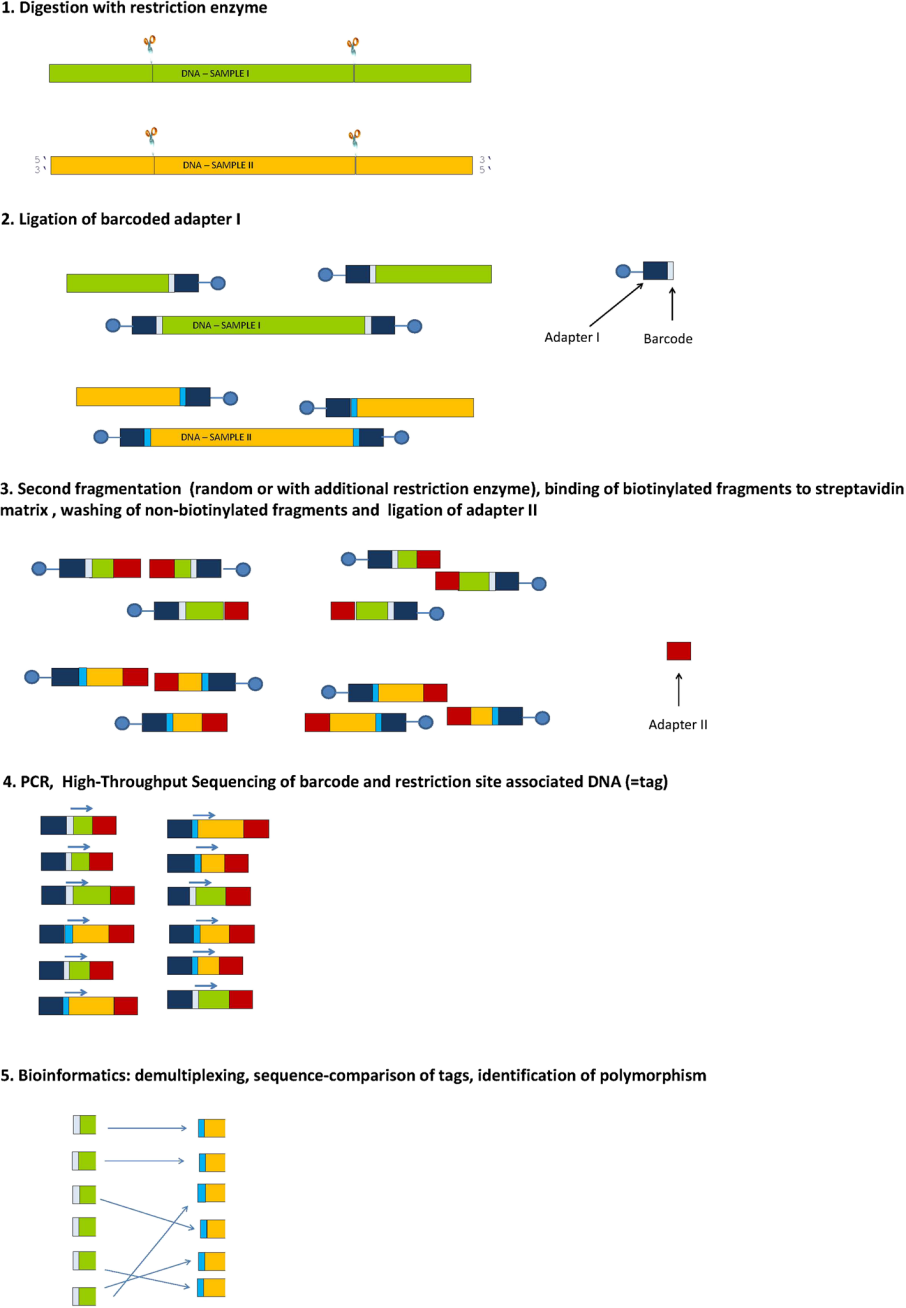
**Schematic outline of a typical “Restriction Enzyme Anchored Sequencing” (REAS) approach.** (1) DNA from different genotypes is digested with one or several restriction enzymes. (2) Biotinylated adapters that carry a barcode for distinguishing the different genotypes are ligated to the restriction site. (3) A second fragmentation step can be applied, either involving random shearing, or further digestion of the sample with a second restriction enzyme. Biotinylated fragments are then bound to a streptavidin matrix. A second adapter is ligated to the construct that is now ready for sequencing, usually after some PCR cycles. A size selection-step can be included after the first or the second restriction digestion to further reduce the complexity. A mix of both adapters or Y-adapters can also be ligated directly after the first or second digestion or after random shearing. (4) Samples are sequenced and (5) sorted according to barcode, and the tag-sequences are compared for single nucleotide polymorphism detection.

Several strategies were developed that aim to enrich particular portions of the genome prior to sequencing. One obvious approach is to sequence only the transcriptome that is available in ESTs or cDNA libraries (for details see "Transcriptome sequencing: RNAseq and related approaches" below). Other techniques rely on the prior amplification of certain genomic subsets using PCR with selected primers (for example, with SRAP primers [321]), or enrich certain parts of the genome by hybridization with a pre-cast set of oligonucleotides (for example, see [322]). In the currently most widely used technique, genomic DNA is first digested with a restriction enzyme, and only the regions on both flanking sites of the enzyme-recognition sites are sequenced (for details see "Restriction Enzyme Anchored Sequencing: RADseq and related approaches" below). This principle was first presented by Altshuler and colleagues [325] who described it as “reduced representation shotgun sequencing”, still using Sanger methodology. A different version of this approach, already involving high-throughput sequencing, was introduced by van Orsouw and colleagues [323] who referred to their technique as CRoPS (Complexity Reduction of Polymorphic Sequences). The most widely used term for the concept, including its many variants, was however suggested by Baird and colleagues [319] who coined their approach “Restriction Site Associated DNA Sequencing” (RADseq). As all of these techniques aim at sequencing restriction site-associated DNA, we will here use the more general term “Restriction Enzyme Anchored Sequencing” (REAS; see Figure 4).

#### Restriction Enzyme Anchored Sequencing: RADseq and related approaches

Depending on its recognition sequence, any given restriction enzyme targets only a small proportion of the entire genome. Restriction digestion is therefore the first (and sometimes the only) step of reducing genomic complexity in REAS. As the restriction sites in genomes of closely related genotypes are usually shared, orthologous genetic regions are sequenced throughout the different genotypes. By choosing an appropriate (pair of) restriction enzyme(s), the extent of reduction of genome representation can be tailored experimentally. For example, a four-base cutter would (in a theoretical genome with a random base distribution) cut every 256 bp, and a six-base cutter every 4,096 bp. In order to focus the sequencing on the non-repetitive, transcriptionally active parts of the genome, most applications make use of methylation-sensitive enzymes that digest only non-methylated sites. Although different epigenetic regulation and accompanying methylation patterns might occur in the different genotypes of interest and hence there is the risk of sequencing and comparing different genetic regions in different genotypes, the advantages outweigh this risk.

One version of the many different REAS techniques is summarized in Figure 4, as a representative example. After digestion, an adapter is ligated to the restriction site, which can directly be used for sequencing on the desired high-throughput platform. The adapter contains a sample-specific barcode sequence which permits the simultaneous “multiplexed” high-throughput sequencing of numerous samples. The adapter can be biotinylated at the opposite end of the ligation site (as in Figure 4) or contain other modifications that inhibit concatemerization of the adapters. After barcoding, the DNA can be either further digested with another restriction enzyme, as in “Double Digest RADseq” [326] or can be randomly sheared to reach the optimal size for the desired sequencing platform. The latter approach was chosen in the original RADseq paper [319]. A second, platform-specific adapter is then ligated to the opposite end of the adapter-DNA fragment. A further reduction of complexity can be achieved by size-selection of the digested DNA. In this case, a frequently cutting restriction enzyme is used, as was proposed by Elshire and colleagues [320] who called their technique “genotyping-by-sequencing” and by Andolfatto and colleagues [318], who used the term “multiplexed shotgun genotyping”.

The primary data comprise the sequence information of the region flanking the restriction site to which the adapters were ligated. These restriction site-associated DNA sequences are referred to as “tags”, or “RAD tags”. Sequencing can be performed either by single end- or by paired-end sequencing. The latter is usually more cost efficient and facilitates mapping of the sequenced region to known related genomes [327,328]. By far the largest part of the restriction sites will usually represent orthologous loci throughout the different genomes. The tagged sequences can then directly be aligned and compared, allowing for the identification of SNPs or indels. The RAD sequencing approach is different from AFLPs or CAPS markers, which focus on differences in the recognition sites themselves. The genome space that is analyzed by an REAS is hence much bigger as compared to procedures that are based on restriction-site polymorphisms only. However, absence versus presence of restriction sites in the different organisms can also be exploited.

Provided that the genome under investigation is not too large, and an adequate sequence coverage is obtained, each particular RAD tag is resequenced many times. The data obtained from an REAS experiment can therefore be exploited for identifying huge numbers of SNP markers [329,330]. REAS data can also be directly used for generating linkage maps from a segregating population [331], or the polymorphic DNA sequences can be annotated to a reference genome, for genomic localization of the newly identified markers [92]. The RAD tags can also be annotated to genomes of plants with high levels of synteny as a cross-reference. This is a big advantage compared to many other DNA fingerprinting techniques, as, when a reference genome is available, the polymorphisms can often directly be localized, which facilitates, for example, fine mapping of QTL. The newly discovered SNPs and other polymorphisms can also be used as markers for fingerprinting or mapping in subsequent steps - for example, in microarrays or CAPS [330]. Several programs are available for the analyses of the data, such as “RADtools”, inaugurated by Baxter and colleagues [332], “Stacks” [333], “UNEAK” [334], “TASSEL” [335] and “Rainbow” [336]. To determine points of chromosomal recombination, a Hidden Markov Model was implemented by Andolfatto and colleagues [318].

Numerous applications of REAS have recently been described in plants (see the review by Poland and Rife [337]). For example, in artichoke, *Cynara cardunculus*, Scaglione and colleagues [330] discovered approximately 34,000 SNPs and nearly 800 indels from 9.7 million reads, corresponding to 1,000 Mb of sequence. Bus and colleagues [329] sequenced more than 113,000 RAD tags and identified more than 20,000 SNPs and indels in the allotetraploid rapeseed, *Brassica napus*. Yang and colleagues [338] used RADseq in a BSA to identify markers for anthracnose disease resistance in *Lupinus angustifolius*. Finally, Pfender and colleagues [331] used RADseq to genotype 193 F1 individuals from a cross between stem-rust resistant and susceptible parental lines of *Lolium perenne* and found several major QTLs for rust resistance.

#### Transcriptome sequencing: RNAseq and related approaches

Another widely used application of high-throughput sequencing for the discovery of polymorphisms is the sequencing of cDNA in an approach coined RNAseq (sequencing of transcribed regions). RNAseq is basically EST sequencing without prior cloning and on high-throughput sequencing machines. The technique and its applications in plants were recently reviewed by Martin and colleagues [88], and an “RNAseq tutorial” was presented by Wolf [339]. Several advantages make transcriptome sequencing very appealing. (1) After *de novo* assembly or annotation, the protein sequence as well as its variants are directly revealed. These polymorphisms represent the ideal markers for gene identification in a mapping approach, as the gene itself and not a co-segregating, anonymous genomic region is marked. (2) The method reduces the complexity of a genome to its transcriptionally active parts and thus allows the comparison of very large genomes. (3) RNAseq simultaneously allows both the determination of a genotype, and the assessment of quantitative gene expression, which permits the analysis of expression QTLs. This concept was described by Harper and colleagues [340] as “associative transcriptomics”. These authors used transcriptome sequencing in *Brassica napus* to correlate differences in gene sequences and gene expression on the one hand with trait variation on the other. The downside of the RNAseq approach is that, in order to be able to sequence a transcript, it must be expressed under the given circumstances - that is, in the investigated tissue - and at the time point of RNA isolation. Another problem is the uneven distribution of the transcript species, which either requires very deep sequencing, or normalization prior to sequencing. This problem especially concerns rare transcripts, such as those coding for transcription factors or other regulatory elements.

RNAseq has also been used in BSA. For example, Trick and colleagues [341] fine-mapped a cloned grain protein content gene, GPC-B1, in wheat, whereas Liu and colleagues [342] were able to directly locate, identify and clone the long-searched gene for glossy3 (gl3), which proved to be a putative myb transcription factor. A high-throughput sequencing-based, reduced complexity analysis strategy for plant transcriptomes was recently presented by Kahl and colleagues [343] who used an Illumina platform to sequence only the 3′-UTRs of the transcripts with Massive Analysis of cDNA Ends. As 3′-UTRs are the most polymorphic regions of a transcript, they provide a rich source of molecular markers. This strategy allows the sequencing of a larger number of genotypes in a multiplexed assay at sufficient coverage, when compared to RNAseq, but additionally reveals quantitative gene expression values for each transcript. To describe this concept, the term “TranSNiPtomics” was invented [343]. Unlike REAS, the technique does not depend on restriction enzymes, but targets a region of 100 to 500 bp upstream of the poly-A tail of each transcript molecule. The approach was recently successfully applied to fine-map an introgressed gene for yellow dwarf virus disease from *H. bulbosum* into barley [344].

### Outlook

The large-scale identification of SNPs and SSRs via high-throughput sequencing approaches is now becoming routine, and highly multiplexed SNP-based genotyping-by-sequencing methods have already been used for numerous applications in many crop plants and model species. This is especially true for genetic mapping, as has been outlined in Genetic mapping. A steadily growing number of studies is also taking care of non-model wild species, where no reference genome is available [345,346]. In the field of population genetics, for example, the ability to sample the genome at much higher densities than ever before overcomes the limitations of traditional marker methodology, allowing for the first time the evaluation of genetic variation patterns across the whole genome [347,348]. The results and insights gained from such “population genomic” approaches will have an enormous impact on the fields of ecology and conservation, as was highlighted in a recent special issue of *Molecular Ecology* (see [349] and other papers in the same volume). Plant systematists will benefit from what may be called “next generation phylogenetics” [350], since plant phylogenies can now be set up on the basis of fully sequenced plastid genomes [351] and/or multiple sets of nuclear genes [352]. New and potentially useful nuclear markers can be identified by sequence comparisons over large taxonomic distances [353], and direct sequence analysis of barcoded individuals may resolve orthology problems in polyploid phylogenetics [352]. Finally, better-resolved phylogenies will help to elucidate species boundaries and species relationships in recent radiations (see the review by Harrison and Kidner [354]).

Where do we go from here? Most certainly, the full technological capabilities are still far from being attained, and “next-next-generation” single-molecule sequencing is currently replacing some of the pioneering methods such as 454 and SOLiD, which are already on the brink of extinction. Further improvements and innovations of sequencing methodology can be expected, and will soon allow for cost-effective genotyping-by-sequencing of any non-model plant species. Even whole-genome sequencing might become feasible for certain applications, such as the analyses of somaclonals or mutation screening. However, obtaining complete genomic sequence data for all individuals included in a study would be unnecessary in most situations, and only inflate the costs. The tremendously huge data sets generated by next-generation sequencing need to be handled and stored, and bioinformatics has already become a real challenge. An important question will therefore have to be asked at the onset of each study - that is, how to allocate the finite number of resources between (1) sequence coverage (that is, the number of times that a particular nucleotide is sequenced), (2) sampling density (that is, the number of individuals analyzed), and (3) the fraction of the genome that is subjected to sequencing [355]. We envisage that sequencing efforts will normally be confined to the necessary minimum also in the future, and that DNA fingerprints generated with an adequately tailored set of markers, obtained by low coverage sequencing of a well-selected representation of a genome, will remain the method of choice for most application areas in plant genotype identification, population genetics, relatedness studies and mapping. We therefore expect that the term “DNA fingerprinting”, once created by Alec Jeffreys for describing the unequivocal identification of human individuals by minisatellite hybridization, will survive in the long run, even in the era of brute force DNA sequencing.

## Abbreviations

AFLP: Amplified fragment length polymorphism; AM: Association mapping; ASP: Allele-specific PCR; bp: Basepairs; BSA: Bulked Segregant Analysis; CAPS: Cleaved amplified polymorphic sequences; cDNA: copied DNA; cM: centiMorgan; cpDNA: chloroplast DNA; cpSSR: chloroplast simple sequence repeat; CRoPS: Complexity Reduction of Polymorphic Sequences; DAMD: Direct amplification of minisatellite DNA; DArT: Diversity Arrays Technology; EST: Expressed sequence tag; FIASCO: Fast isolation by AFLP of sequences containing repeats; GS: Genomic selection; GWS: Genome-wide selection; HRM: High-resolution melting; IBD: Isolation-by-distance; IRAP: Inter-retrotransposon amplified polymorphism; ISSR: Inter-simple sequence repeat; ITS: Internal transcribed spacer; LD: Linkage Disequilibrium; LM: Linkage mapping; LTR: Long terminal repeat; MAC-PR: Microsatellite DNA allele counting - peak ratios; MADCE: Microsatellite allele dose and configuration establishment; MAS: Marker-assisted selection; mtDNA: Mitochondrial DNA; NAS: Nested association mapping; PCR: Polymerase chain reaction; PDO: Protected denomination of origin; QTL: Quantitative trait loci; RAD: Restriction Site Associated DNA; RADseq: Restriction Site Associated DNA Sequencing; RAPD: Random amplified polymorphic DNA; rDNA: Ribosomal DNA; REAS: Restriction Enzyme Anchored Sequencing; REMAP: Retrotransposon-microsatellite amplified polymorphism; RFLP: Restriction fragment length polymorphism; RGAP: Resistance gene-analog polymorphism; RIL: Recombinant Inbred Line; RNAseq: Sequencing of transcribed regions; SAMPL: Selective amplification of polymorphic microsatellite loci; SCAR: Sequence characterized amplified region; SGS: Spatial genetic structure; SNP: Single nucleotide polymorphism; SRAP: Sequence-related amplified polymorphism; S-SAP: Sequence-specific amplification polymorphism; SSR: Simple sequence repeat; STR: Synthetic tandem repeat; TRAP: Target region amplification polymorphism; UTR: Untranslated region; WGS: Whole genome sequencing.

## Competing interests

The authors declare that they have no competing interests.

## Authors’ contributions

HN, KW and BR participated in the writing of the manuscript. All authors read and approved the final manuscript.
